# Development of STEAP1 targeting chimeric antigen receptor for adoptive cell therapy against cancer

**DOI:** 10.1016/j.omto.2022.06.007

**Published:** 2022-06-22

**Authors:** Yixin Jin, Kristina Berg Lorvik, Yang Jin, Carole Beck, Adam Sike, Irene Persiconi, Emilie Kvaløy, Fahri Saatcioglu, Claire Dunn, Jon Amund Kyte

**Affiliations:** 1 Department of Cancer Immunology, Institute for Cancer Research, Radiumhospitalet, Oslo University Hospital, Mail Box 4950 Nydalen, 0424 Oslo, Norway; 2 Institute for Cancer Genetics and Informatics, Oslo University Hospital, Oslo, Norway; 3 Department of Biosciences, University of Oslo, Oslo, Norway; 4 Department of Clinical Cancer Research, Oslo University Hospital, Oslo, Norway

**Keywords:** chimeric antigen receptor, cell therapy, prostate cancer, STEAP1, cancer immunotherapy, metastatic cancer mouse model, CAR T cell

## Abstract

Chimeric antigen receptors (CARs) that retarget T cells against CD19 show clinical efficacy against B cell malignancies. Here, we describe the development of a CAR against the six-transmembrane epithelial antigen of prostate-1 (STEAP1), which is expressed in ∼90% of prostate cancers, and subgroups of other malignancies. STEAP1 is an attractive target, as it is associated with tumor invasiveness and progression and only expressed at low levels in normal tissues, apart from the non-vital prostate gland. We identified the antibody coding sequences from a hybridoma and designed a CAR that is efficiently expressed in primary T cells. The T cells acquired the desired anti-STEAP1 specificity, with a polyfunctional response including production of multiple cytokines, proliferation, and the killing of cancer cells. The response was observed for both CD4^+^ and CD8^+^ T cells, and against all STEAP1^+^ target cell lines tested. We evaluated the *in vivo* CAR T activity in both subcutaneous and metastatic xenograft mouse models of prostate cancer. Here, the CAR T cells infiltrated tumors and significantly inhibited tumor growth and extended survival in a STEAP1-dependent manner. We conclude that the STEAP1 CAR exhibits potent *in vitro* and *in vivo* functionality and can be further developed toward potential clinical use.

## Graphical abstract

**Figure:**
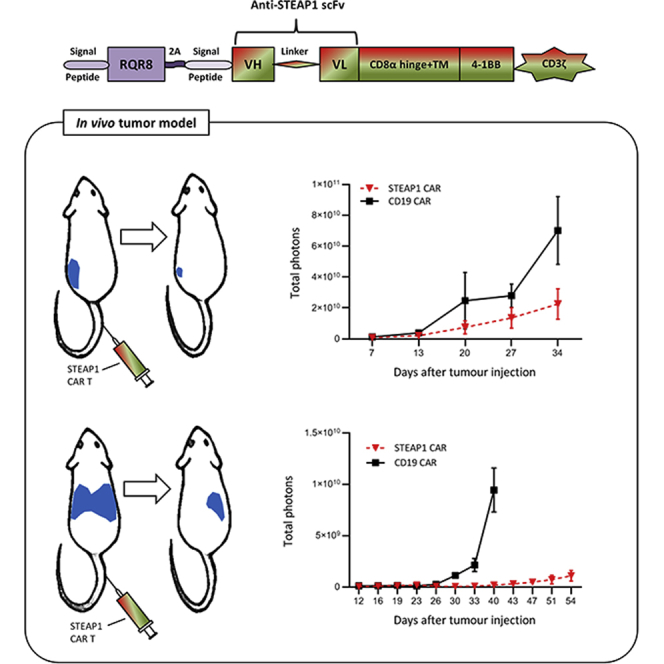

## Teaser

Jin et al. have developed a CAR targeting STEAP1 for use in cell therapy against cancer. They demonstrate that the CAR confers T cells with potent and specific anti-tumor activity *in vitro* and in two mouse models of prostate cancer. The CAR may be applied against different cancers expressing STEAP1.

## Introduction

Immunotherapy has emerged as a highly effective approach for cancer treatment. An important breakthrough has been the use of genetically engineered T cells that are retargeted to kill cancer cells through the use of chimeric antigen receptors (CARs). This therapy has shown remarkable efficacy against advanced B cell leukemia and lymphoma, leading to approvals for use in the clinic.^1^^,^^2^ In most CAR constructs, the antigen-binding part consists of a single chain fragment variable (scFv) derived from a monoclonal antibody (mAb). This domain is fused to a spacer, a transmembrane domain and an intracellular signaling domain from the T cell receptor (TCR) complex. Second-generation CAR constructs also include a signaling domain from a co-stimulatory molecule (e.g., CD28 or 4-1BB).^3^ This co-stimulatory domain confers the T cells with more potent effector functions, and the inclusion of such a domain has proved crucial to the clinical efficacy of the CARs.^4^, ^5^, ^6^ In contrast to TCRs, a CAR binds independent of human leukocyte antigen (HLA) and may thus be used across the entire patient population.

Prostate cancer is the most common cancer among males.^7^ Localized prostate cancer can be eliminated by surgery or radiotherapy, whereas metastatic disease is treated with androgen ablation therapy. Most metastatic patients eventually develop castration-resistant prostate cancer. At this point, available treatment options are limited and only extend survival by a few months, giving a median survival of about 13 months.^8^ Studies with immune checkpoint inhibitors have so far not shown efficacy against prostate cancer. This fact probably relates to factors intrinsic to prostate cancer, such as a low mutational burden. CAR T cell therapy may overcome these obstacles and is considered an interesting option.^9^^,^^10^ It is, however, important to target a CAR antigen that is retained in the more aggressive cancers and less likely to be lost during tumor progression.^11^

There are promising clinical data for CAR T cell therapy against myeloma^12^ and a few other non-B cell cancers, but no breakthrough has yet been achieved for this therapy against solid tumors.^13^ This fact points to possible hurdles for CAR T cell therapy in solid tumors, but also suggests the importance of identifying a good target antigen. Of note, CD19, which is targeted in most approved CAR therapies, is not a tumor-associated antigen but a normal tissue differentiation antigen expressed both by malignant and normal B cells. The success of CAR therapy against B cell malignancies is related to the fact that B cells are dispensable, and that CD19 expression is relatively conserved among malignant B cells. Immunosuppression, T cell homing issues, and other factors may represent important hurdles for CAR T therapy in solid tumors. However, the first effective adoptive cell therapy in cancer was achieved with tumor-infiltrating lymphocytes against a solid tumor, malignant melanoma.^14^^,^^15^ It is thus clear that the potential of CAR T cell therapy against solid cancers can only be evaluated when a CAR against an appropriate target antigen is available.

We have developed a CAR that binds to the 339-amino-acid cell surface protein six-transmembrane epithelial antigen of prostate-1 (STEAP1).^16^^,^^17^ STEAP1 is expressed in ∼90% of prostate cancers and in considerable subpopulations of many other cancer types, such as lung cancer, bladder cancer, Ewing sarcoma, breast cancer, pancreatic cancer, glioblastoma, ovarian cancer, leukemia, lymphoma, and head and neck cancer.^17^, ^18^, ^19^ In normal tissues, STEAP1 is mainly expressed in the prostate, which is not a vital organ and can be removed in patients with prostate cancer. Some reports also indicate a low to moderate STEAP1 expression in other normal tissues, such as adipose tissue, breast, and bladder.^17^, ^18^, ^19^ However, across different studies, a consistently high normal tissue expression of STEAP1 has only been documented in prostate tissue. For other normal tissues, STEAP1 has mainly been detected by mRNA expression and the findings have been inconsistent.^17^, ^18^, ^19^ Overall, these data suggest that the STEAP1 protein expression and the toxicity of STEAP1-targeted therapies in different tissues are likely to be limited. Interestingly, the first published trial with a STEAP1-targeting antibody-drug conjugate indicated a favorable safety profile.^20^ Moreover, the side effects in this trial appeared related to the chemotherapy payload rather than to STEAP1-directed toxicity.

The exact biological function of STEAP1 has not yet been determined, but it is thought to play a role in cell adhesion and intracellular communication.^16^^,^^17^ Across multiple cancer forms, STEAP1 has been associated with tumor proliferation, progression, and invasiveness.^17^^,^^18^^,^^21^ In prostate cancer, animal studies have shown that STEAP1 knockdown counters androgen actions, inhibits proliferation, and induces apoptosis in tumor cells.^22^ In cancer forms where peritoneal metastases represent a key development, several studies have indicated that STEAP1 promotes tumor invasion into the peritoneum.^23^, ^24^, ^25^

CAR T cell therapy is resource demanding and will most likely be clinically applicable only for metastatic disease in solid cancers. Since STEAP1 has been associated with increased risk of prostate cancer relapse and with a high Gleason score,^26^^,^^27^ this has led to interest in using STEAP1 detection for monitoring metastatic disease^28^ as well as making it an attractive CAR target in metastatic prostate cancer. In addition, a STEAP1 CAR may be used against other STEAP1 positive cancers.^17^^,^^18^^,^^21^^,^^23^, ^24^, ^25^ Here, we report the development of a CAR against STEAP1 and show that it has significant efficacy against prostate cancer cells *in vitro* and *in vivo*.

## Results

### Characterization of STEAP1 antibody and cloning of STEAP1 CAR

**Figure 1:**
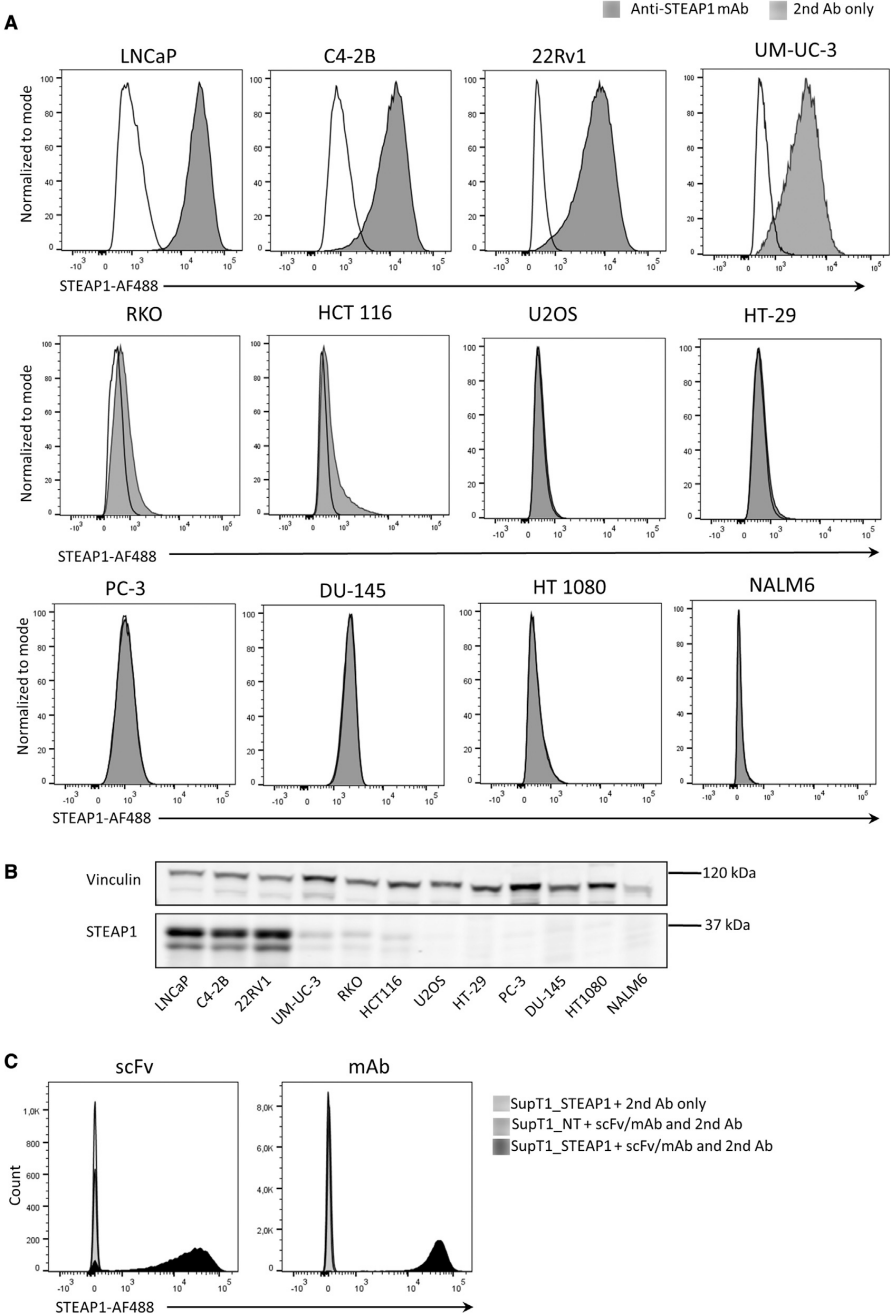
STEAP1-specific staining by mAb and scFv (A) A selection of six STEAP1-positive (LNCaP, C4-2B, 22Rv1, UM-UC-3, RKO, and HCT 116) and six STEAP1-negative cancer cell lines were stained with the anti-STEAP1 mAb Oslo-1 and a secondary antibody (Ab) (goat anti-mouse IgG AF488), and analyzed by flow cytometry. Gray filled histograms, anti-STEAP1 mAb + secondary (second) Ab; black open histograms, second Ab control. (B) Western blot analysis of STEAP1 expression on cell lines using a different anti-STEAP1 antibody (sc-10262). (C) STEAP1 transduced SupT1 cells (SupT1_STEAP1) and non-transduced SupT1 cells (SupT1_NT) were stained with the Oslo1 scFv (left) or the corresponding mAb (right) and analyzed by flow cytometry. Overlays display SupT1_STEAP1 cells stained with scFv/mAb +second Ab (black filled) or only with the second Ab (black open), or SupT1_NT cells stained with scFv/mAb +second Ab (light gray filled).

To develop a CAR that binds the tumor-associated antigen STEAP1, we first established a panel of STEAP1-positive and -negative cancer cell lines and used these for screening a series of supernatants from different hybridomas (data not shown). We found that the supernatant from ATCC PTA-5803 (X120.545.1.1) was highly STEAP1 specific, in line with reported data,^29^ and selected this hybridoma as a basis for CAR development. We then identified the Ab-coding sequences of the hybridoma. To confirm that the sequences were correct, we generated a synthetic mAb, called Oslo-1, incorporating the identified binding sequences, and tested if this mAb would bind to STEAP1 by staining a panel of tumor cell lines for flow cytometry. As shown in Figure 1A, the Oslo-1 mAb specifically bound to STEAP1. The four prostate and bladder cancer cell lines LNCaP, C4-2B, 22Rv1, and UM-UC-3, which are all known to express STEAP1,^17^^,^^22^^,^^29^^,^^30^ displayed robust mAb staining. Two colon cancer cell lines, RKO and HCT116, stained moderately positive with the synthesized Ab. Further, the specificity was confirmed by a lack of staining in PC-3 and DU-145 (prostate cancer), HT1080 (fibrosarcoma), U2OS (bone osteosarcoma), HT-29 (colorectal adenocarcinoma), and NALM6 (acute lymphoblastic leukemia) (Figure 1A). The STEAP1-expression in the cell lines were confirmed by western blot, using a different anti-STEAP1 antibody (Figure 1B). We then designed an scFv based on the VL and VH sequences, for use as an antigen-binding unit in a CAR. It is well known that in some cases the scFv does not retain the specificity and other binding properties of the mAb. To determine if the scFv, termed Oslo1, displayed a similar binding profile to the parental mAb, we generated a construct in which the scFv is fused to an immunoglobulin tail sequence and assessed binding to ectopically expressed STEAP1 on SupT1 cells. Non-transduced (NT) SupT1 cells were used as a negative control. Flow cytometry analysis showed that, like the parental Ab, the Oslo1 scFv specifically bound to STEAP1 (Figure 1C).

**Figure 2:**
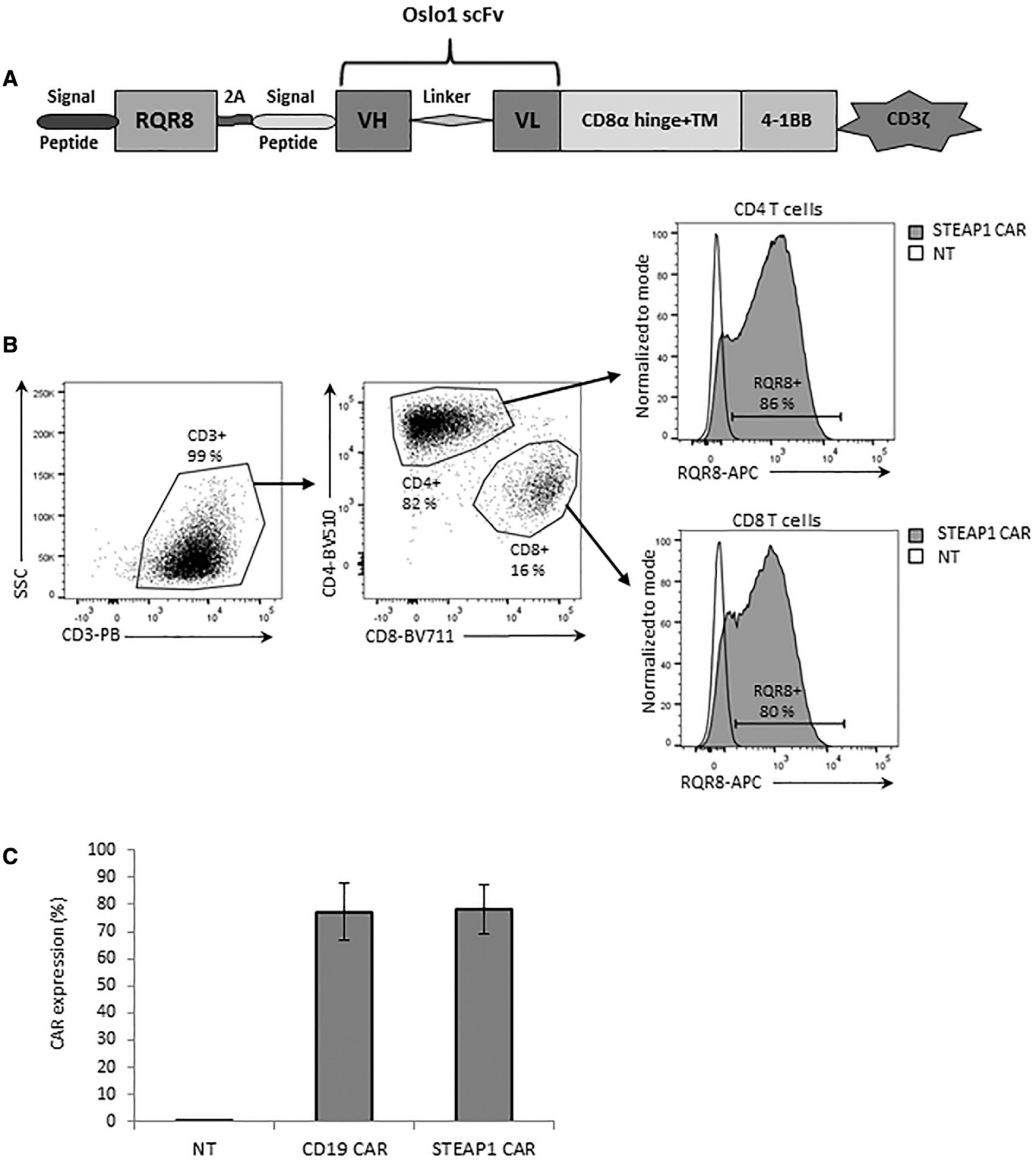
STEAP1 CAR design and expression (A) Schematic of STEAP1 CAR from N terminus to C terminus: signal peptide, RQR8, 2A self-cleavage peptide, signal peptide for the CAR, Oslo1 scFv targeting STEAP1, CD8a hinge domain, CD8a transmembrane domain, 4-1BB co-stimulatory domain, and CD3ζ intracellular signaling domain. (B) T cells were surface stained for CD3, CD4, and CD8 (left and middle) and analyzed by flow cytometry. CAR expression was identified by RQR8 staining. Histograms to the right show STEAP1 CAR-transduced (gray filled) CD4+ and CD8+ T cells, overlaid with NT T cells (black open). (C) The expression of the STEAP1 CAR and a CD19-specific CAR with an identical backbone was measured in transduced T cells from 10 healthy donors. Error bars indicate SD.

To develop a CAR, we generated a construct in the retroviral vector SFG that incorporated the Oslo1 scFv fused to a CD8a spacer and transmembrane (TM) domain, an intracellular CD3ζ domain, and a 4-1BB co-stimulatory domain (Figure 2A). The suicide/sorter/marker gene RQR8^31^ was cloned into the SFG vector in tandem with the CAR construct. RQR8 is a compact gene combining minimal target epitopes from CD34 and CD20.^31^ RQR8 allows for large-scale purification of transduced T cells with the clinically approved CliniMACS CD34 system, and the elimination of transduced cells with rituximab. Here, we use it as a marker to identify the expression of the CAR construct.

### STEAP1 CAR expression and phenotype of CAR T cells

STEAP1 CAR expression was assessed by flow cytometry on primary T cells that were obtained from 10 different donors. The STEAP1 CAR was highly expressed on both CD4^+^ and CD8^+^ T cells, with a transduction efficacy in primary T cells of 60%–88% (Figure 2B). Among the 10 donors, the average STEAP1 CAR expression was 78% (Figure 2C).

The phenotype of the peripheral blood mononuclear cell (PBMC)-derived CAR-transduced cells was characterized by flow cytometry. Figure S1 shows the characterization of CAR T cell cultures from four healthy donors, each tested in duplicate, performed 7 days after initial PBMC stimulation. At this time point, there was generally >90% T cells, while the fraction of CD4 and CD8 cells varied between the donors (data not shown). More than 95% of CAR T cells were central memory (CM) or effector memory (EM) cells. The CM population was slightly increased in CAR T cells, compared with NT controls (Figure S1A). About 70%–80% of the T cells were CD25^+^ at day 7, but most CD4+ T cells remained negative for the activation/exhaustion makers PD1, TIGIT, LAG3, and TIM3. The CD8^+^ T cells expressed more LAG3 and TIM3, but were mostly negative for PD1 (Figures S1B–S1C). The T cell yield after transduction and expansion varied between donors, but was similar for STEAP1 and CD19 CAR T cells, with a 2- to 5-fold increase in the number of live cells 7 days after initial PBMC stimulation (data not shown).

### Assessment of STEAP1 expression in target cells and generation of STEAP1 knockdown lines

We assessed STEAP1 expression in cancer cell lines by quantitative polymerase chain reaction (qPCR), western blot, and flow cytometry (Figure S2). The results confirmed that the prostate cancer cell lines 22Rv1, LNCaP, and C4-2B are STEAP1 positive. A partial loss of STEAP1 expression was observed in 22Rv1 cells during long-term culture (data not shown). To obtain suitable target cells for evaluation of the STEAP1 CAR, we knocked down STEAP1 expression in LNCaP and C4-2B cells with two different short hairpin RNAs (shRNAs). An shRNA targeting green fluorescence protein (GFP) served as a sham control. As shown in Figure S2, the knockdown resulted in about 65%–80% reduction in STEAP1 mRNA expression, and a similar reduction of protein level as measured by western blot. STEAP1 expression was not detectable by flow cytometry in any of the four knockdown cell lines, using the mAb corresponding to the Oslo1 scFv (Figure S2C).

### CAR T cells exhibit STEAP1-specific functionality *in vitro*

**Figure 3:**
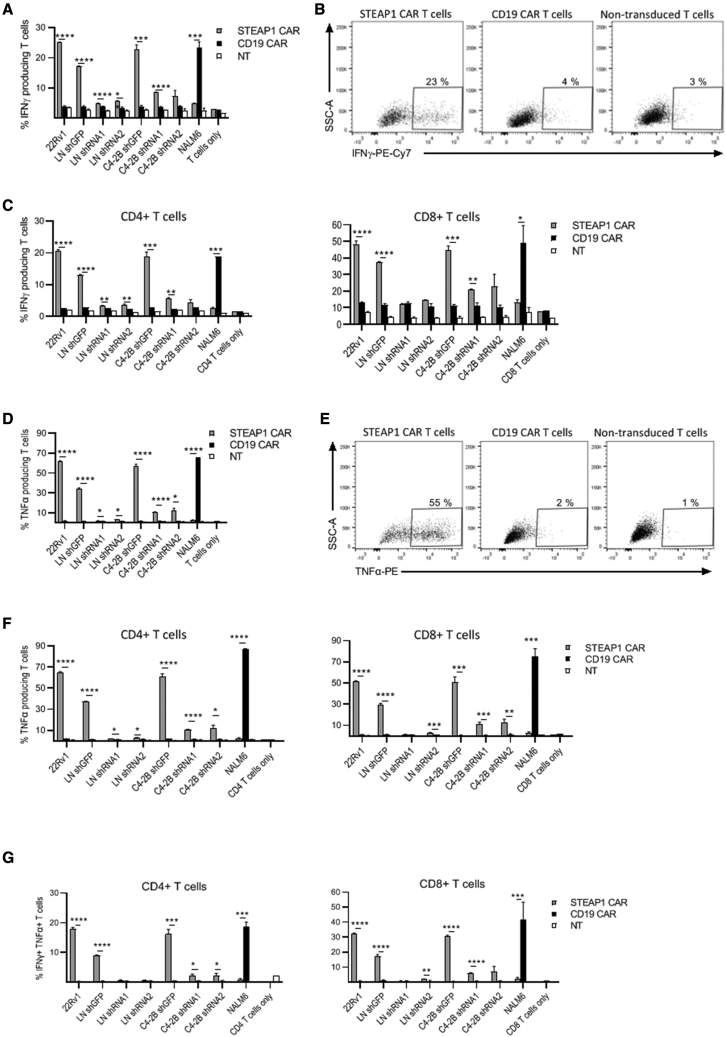
STEAP1-specific production of IFNγ and TNFα by CAR T cells Flow cytometry analysis of STEAP1 CAR T cells (gray bars), CD19 CAR T cells (black bars), or NT T cells (white bars) cultured with different target cells at an effector-to-target (E:T) ratio of 1:3 for 16 h. (A) IFNγ production. (B) Dot plots of T cells co-cultured with C4-2B cells, showing the applied gating. (C) IFNγ production in CD4+ (left) or CD8+ (right) T cells. (D) TNFα production. (E) Dot plots of T cells co-cultured with C4-2B cells. (F) TNFα production in CD4+ (left) or CD8+ (right) T cells. (G) Percentage of individual T cells producing both TNFα and IFNγ. The T cells were gated on RQR8+ (CAR expression), or CD3+ for the NT cells. 22Rv1, LNCaP (LN), and C4-2B are STEAP1^+^ prostate cancer lines. NALM6 is a STEAP1^−^ CD19^+^ leukemia cell line. LNCaP and C4-2B variants with shRNA knocking down STEAP1 (shRNA1, shRNA2) or control shRNA (shGFP) were used as indicated. Data are mean of triplicates, with error bars representing SEM. The percentage of positive cells recorded for the STEAP1 CAR versus the CD19 CAR was compared by multiple t tests. Statistical significance was determined using the Holm-Sidak method, adjusting for multiple comparisons. Adjusted p values are indicated (∗p < 0.05, ∗∗p < 0.01, ∗∗∗p < 0.001, ∗∗∗∗p < 0.0001). The results are representative of two individual experiments.

Next, we characterized the functionality of the STEAP1 CAR. It is well known that CAR constructs may potentially confer a level of non-specific activation on transduced T cells. To assess CAR antigen specificity, we cloned a CD19-specific scFv into the same CAR backbone as the STEAP1 CAR, with identical spacer, transmembrane, and intracellular domains (CD3ζ and 4-1BB). The chosen CD19-specific scFv is derived from the fmc63 hybridoma.^32^ The control anti-CD19 CAR (CD19 CAR) thus resembles tisagenlecleucel (fmc63scFv-CD8a spacer/TM-41BB-z), which is in clinical use against leukemia.^33^ Primary T cells were transduced with either the STEAP1 CAR, the CD19 CAR, or left NT, and then co-cultured for 16 h with STEAP1-positive or -negative target cells. A representative experiment is shown in Figure 3. Flow cytometry analysis demonstrated that ∼25% of the STEAP1 CAR T cells responded by producing interferon gamma (IFNγ) upon co-culture with STEAP1^+^ target cells, compared with ∼3%–4% of NT T cells and control T cells expressing the non-relevant CD19 CAR (Figures 3A and 3B). The CAR specificity was confirmed by the lack of IFNγ production upon co-culture with STEAP1^-^ NALM6 cells (Figure 3A). We found that the STEAP1 CAR T cells were as good at producing IFNγ as the well-established CD19 CAR T cells when cultured together with their cognate targets (Figure 3A). Both CD4^+^ and CD8^+^ T cells exhibited an IFNγ response (Figure 3C). There were more IFNγ producing CD8^+^ than CD4^+^ T cells (∼50% versus ∼20% IFNγ^+^).

To confirm the specificity of the STEAP1 CAR T cells, and assess their activity against targets expressing very low levels of STEAP1, we co-cultured the CAR T cells with the shRNA knockdown versions of LNCaP and C4-2B. Upon STEAP1 knockdown in LNCaP cells, IFNγ production by STEAP1 CAR T cells was reduced to the level of the CD19 CAR T and NT T cell controls. In a similar experiment with C4-2B cells, there was a slight increase of IFNγ-producing STEAP1 CAR T cells in the co-cultures with C4-2B STEAP1 knockdown cells compared with the CD19 CAR T cell controls (Figures 3A and 3C). This was consistent with the incomplete knockdown of STEAP1 in these cells (Figure S2).

We also analyzed tumor necrosis factor alpha (TNFα) production upon co-culturing of CAR T cells with STEAP1+ targets, and found that up to ∼60% of the T cells produced TNFα in contrast to only ∼2% of CD19 CAR T cells (Figures 3D and 3E). Knockdown of STEAP1 strongly attenuated TNFα production in STEAP1 CAR T cells (Figure 3D). In contrast to the results for IFNγ, there was a slightly higher fraction of CD4^+^ T cells than CD8^+^ T cells producing TNFα (Figure 3F); approximately 60% of CD4^+^ T cells produced TNFα after co-culture with 22Rv1 and C4-2B, compared with 50% of CD8^+^ T cells. Combined production of IFNγ and TNFα was demonstrated for nearly all IFNγ^+^ CD4^+^ T cells, and 50%–70% of the IFNγ^+^ CD8^+^ T cells (Figure 3G).

**Figure 4:**
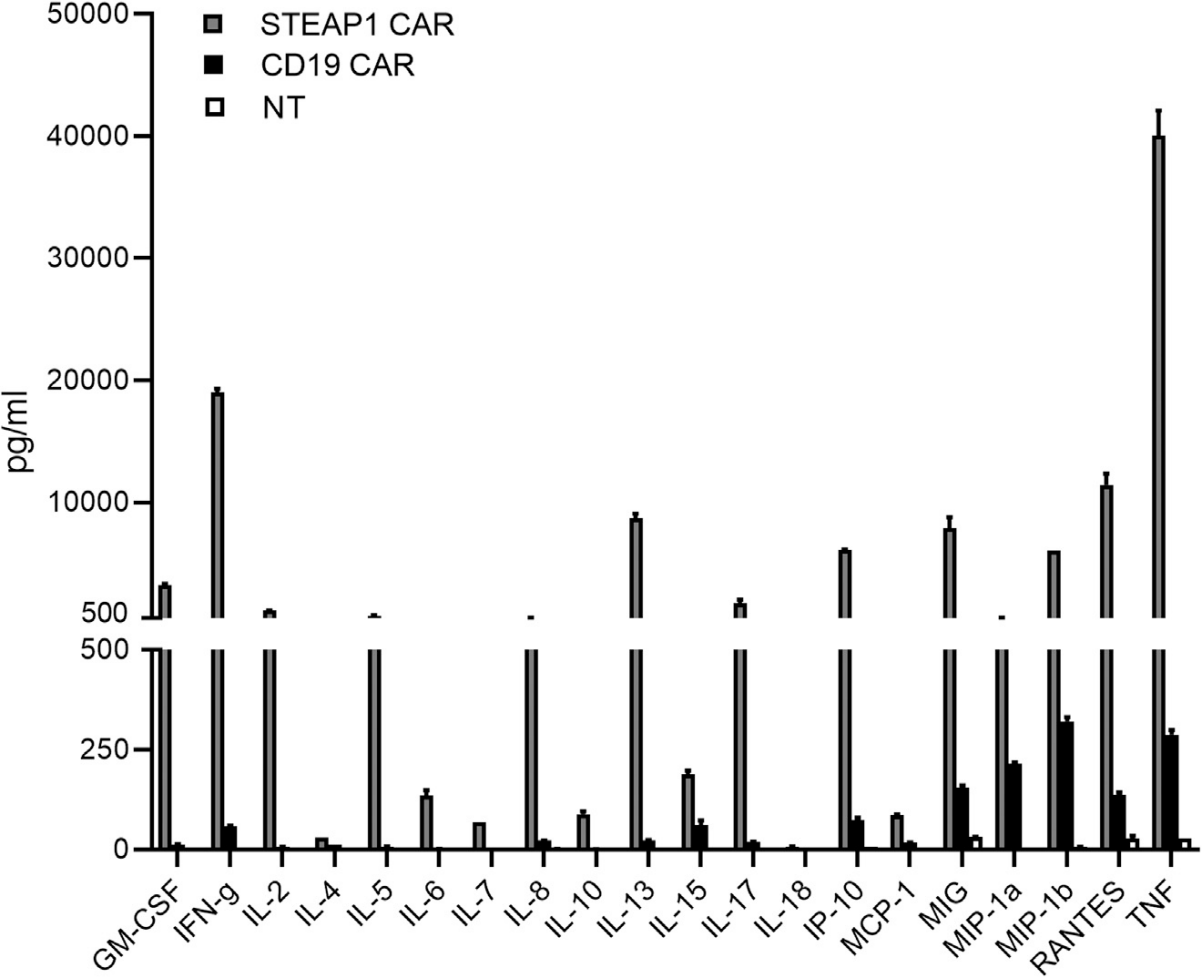
Cytokine profile of T cells co-cultured with STEAP1+ 22Rv1 cells STEAP1 CAR, CD19 CAR, and NT T cells were co-cultured with 22Rv1 cells for 48 h at an E:T ratio of 1:1 and the cell culture supernatant harvested. Twenty different cytokines and chemokines were quantified using the Bio-Plex Multiplex Immunoassay system. Error bars represent SEM from duplicate cultures, each parallel kept separate through T cell stimulation and Bio-Plex assays.

The cytokine profile was further investigated in multiplex assays measuring 20 cytokines. These assays showed that the STEAP1 CAR T cells secreted a wide range of cytokines upon stimulation with 22RV1 cells, while the control T cells (CD19 CAR and NT) did not (Figure 4). The cytokine response was T-helper 1 (Th1) weighted, with high levels of TNFα and IFNγ, and only marginal levels of IL-4 and IL-10, and moderate levels of IL-5. The CAR T cells also secreted substantial levels of the chemokines IP-10, MIG, MIP-1α, MIP-1β and RANTES, suggesting polyfunctionality and a potential to provoke inflammation and attract other immune cells.

To investigate how the CAR T cells proliferated and responded to long-term stimulation from STEAP1^+^ tumor cells, we thawed CAR T cells and performed a 21-day co-culture assay of the T cells with irradiated 22Rv1 cells (Figure S3). The T cells were counted and phenotyped once per week by flow cytometry. The data indicated continued proliferation of the STEAP1 CAR T cells throughout the assay, giving a 9- to 10-fold expansion after 3 weeks (Figure S2A). The proliferation was specific to the STEAP1 CAR, as NT T cells and CD19 CAR T cell controls did not expand. The CD8/CD4 ratio increased about 3-fold for the STEAP1 CAR T cells (Figure S3B). In both CD4+ and CD8+ T cells, the expression of the activation marker CD25 and the checkpoint modulators PD1, LAG3, and TIGIT increased over the 21 days, while TIM3 did not (Figure S3C). The majority of CAR T cells gradually differentiated from a CM to an EM phenotype (Figure S3D).

### STEAP1 CAR T cells kill target cells

**Figure 5:**
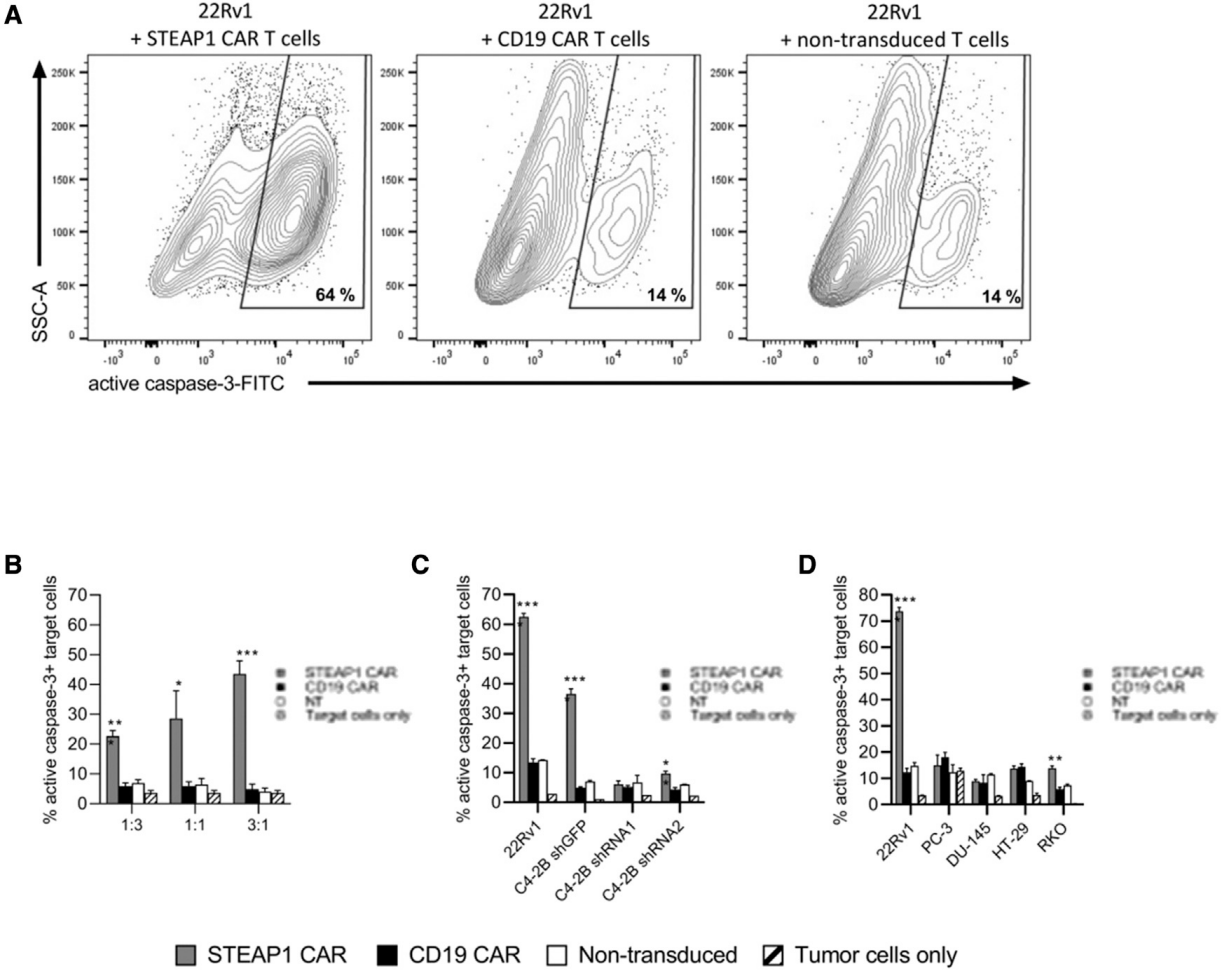
STEAP1 CAR T cells specifically induce apoptosis in STEAP1+ target cells STEAP1 CAR T cells, CD19 CAR T cells, or NT T cells were cultured in triplicates for 16 h with various target cells. Apoptosis of target cells was measured by analyzing the intensity of FITC-DEVD-FMK bound to active caspase-3 by flow cytometry. Target cells cultured alone were included as controls to indicate the baseline level of active caspase-3 in each cell line. (A) Representative contour plots of 22Rv1 cells after co-culture with effector cells, showing gating strategy for identification of cells positive for active caspase-3. (B) Percentage of apoptotic 22Rv1 cells, after co-culture with effector T cells at different E:T ratios, as indicated. (C) Percentage of apoptotic cells among STEAP1-positive and STEAP1-knockdown targets, after co-culture with effector T cells (E:T ratio of 3:1). (D) Percentage of apoptotic cells among STEAP1-positive (22Rv1), STEAP1-negative (PC-3, DU-145 and HT29), or low-level STEAP1 expression (RKO) cell lines, after co-culture with effector T cells (E:T ratio of 3:1). Error bars represent SEM from triplicates. ∗p < 0.05, ∗∗p < 0.01, ∗∗∗p < 0.001. The data are representative for two independent experiments.

We investigated the ability of the CAR T cells to kill tumor cells, by analyzing the induction of active caspase-3 in target cells. STEAP1 CAR T cells, as well as T cells with the irrelevant CD19 CAR and NT T cells, were co-cultured with STEAP1^+^ tumor cells at different effector-to-target (E:T) ratios. Dead cells were excluded with Fixable Viability Dye, and T cells were excluded by staining for CD3 (Figure S4). The induction of active caspase-3 was 5- to 10-fold increased when 22Rv1 cells were cultured together with STEAP1 CAR T cells, compared with CD19 CAR or NT control T cells (Figure 5 and data not shown). The killing increased, as expected, with increasing E:T ratios (Figure 5B). The STEAP1 CAR T cells similarly induced apoptosis in STEAP1^+^ C4-2B cells (Figure 5C). To determine the specificity of the killing, we used the STEAP1 shRNA knockdown C4-2B cell lines described above. Co-culture of STEAP1 CAR T cells with shRNA1- or shRNA2-treated target cells showed a strong decrease in apoptosis induction (Figure 5C). The killing specificity was further confirmed in tests with a panel of prostate and colon cancer cell lines (Figure 5D). Here, we observed specific killing by STEAP1 CAR T cells, compared with control CD19 CAR T cells, of the 22Rv1 target cells, but not of any of the STEAP1-negative targets (PC-3, DU-145, HT-29). A low-level, but still significant, killing by STEAP1 CAR T of RKO cells was detected, in line with the low-level STEAP1 expression observed in this cell line (Figure 1). In all, the cytotoxicity data corresponded with the STEAP1 expression across all cell lines and shRNA knockdown controls.

Next, we investigated the ability of STEAP1 CAR T cells to kill target cells by using the IncuCyte S3 real-time live cell analysis system. This system monitors cell death over time by the continuous imaging the GFP-labeled target cells. As shown in Figure S5, the STEAP1 CAR T cells effectively eliminated the 22Rv1 cells, at both E:T ratio 5:1 and 2.5:1, while CD19 CAR T cell controls only gave limited non-specific effects, similar to NT T cells. The real-time data demonstrated ongoing killing of target cells over several days, only in those exposed to STEAP1 CAR T cells. After 6 days, nearly all target cells were dead (Figures S5A and S5B).

### STEAP1 CAR T cells have robust therapeutic efficacy *in vivo*

To test the *in vivo* functionality of STEAP1 CAR T cells, we established a subcutaneous (s.c.) xenograft mouse model. NOD *scid* gamma (NSG) mice were engrafted s.c. with the human STEAP1-positive prostate cancer cell line 22Rv1 and, once the tumors were palpable, treated with STEAP1 CAR T cells, CD19 CAR T cells, or NT T cells. Tumor growth was monitored by both caliper measurement and bioluminescence imaging. Seven days after tumor cell injection, tumors with a diameter of 3–5 mm could be detected in all mice. At days 9 and 14, the mice were treated with STEAP1 CAR T cells, CD19 CAR T cells, or NT T cells, injected intravenously (i.v.). In addition, the mice received rhIL-2 twice a week. The 22Rv1 cells were confirmed not to express CD19 (Figure S6A). The mAb on which our STEAP1 CAR is based does not recognize murine STEAP1, as assessed by the TRAMP-C2 cell line, which has been reported to express murine STEAP1^34^ (Figure S6B).

**Figure 6:**
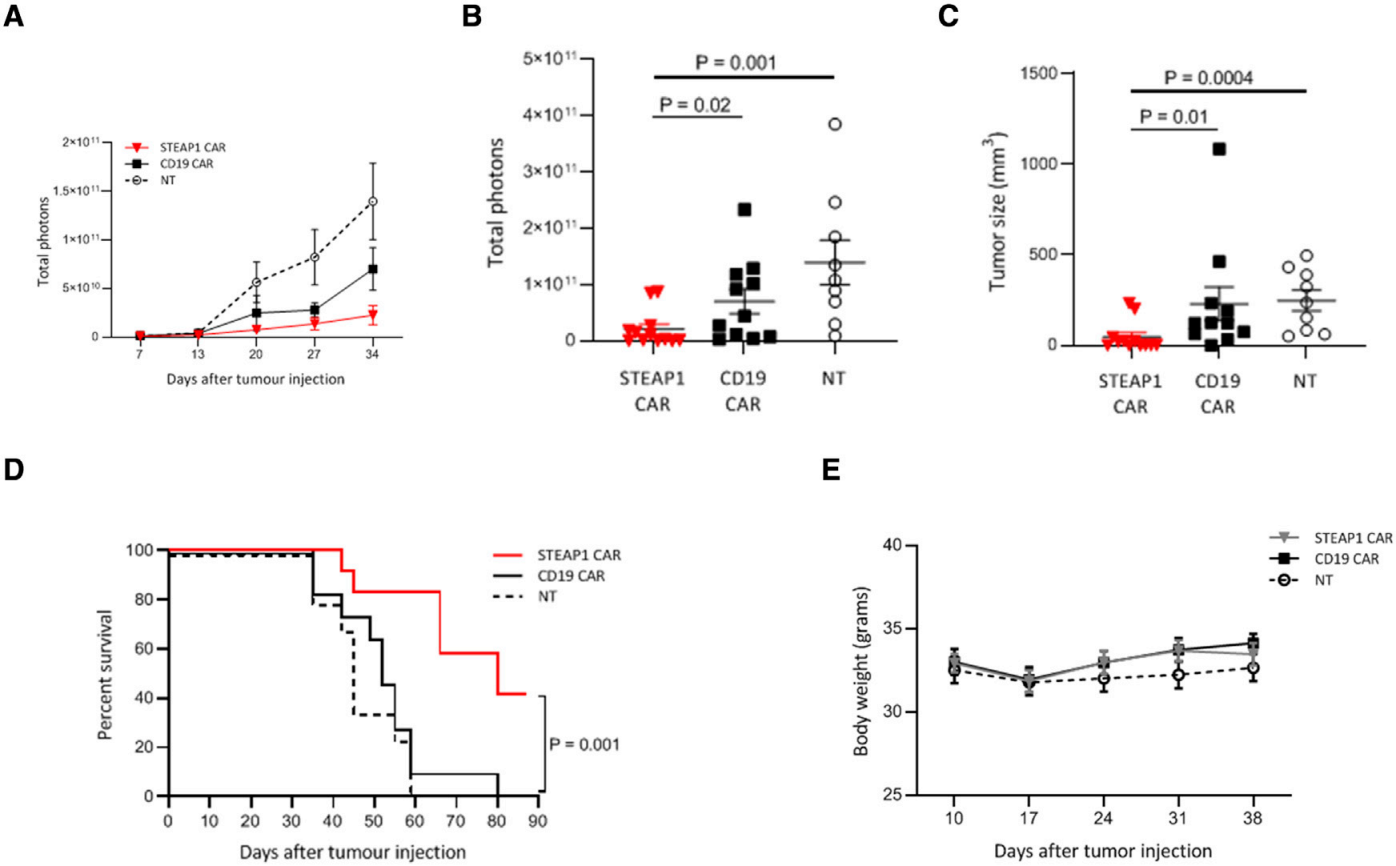
STEAP1 CAR T cells inhibit tumor growth *in vivo* NSG mice were engrafted with 2 × 10^6^ luciferase-expressing 22Rv1 prostate cancer cells subcutaneously on the hind leg. On day 9 and 14, the mice were treated with 1 × 10^7^ STEAP1 CAR T cells (N = 12), CD19 CAR T cells (N = 11), or NT T cells (N = 9) by i.v. injection, and 100 IU/g body weight rhIL-2 was given intraperitoneally twice a week. (A) Tumor growth as measured once a week by bioluminescence IVIS imaging. The data are presented as mean ± SEM in each group of mice. (B) Bioluminescence signals (mean ± SEM) 34 days after tumor engraftment. (C) Tumor size (mean ± SEM) 33 days after tumor engraftment. The length, width, and depth of the tumors were measured with a caliper. Statistical analyses in (B) and (C) were performed with Mann-Whitney U test. (D) Bioluminescence signals for each individual mouse at day 34. (E) Kaplan-Meier plot showing extended survival for the mice treated with the STEAP1 CAR T cells (red line), compared with CD19 CAR T cells (black line) or NT T cells (dotted line). The mice were euthanized when tumors reached 1,000 mm^3^. Statistical analysis was performed using the log rank test. (E) The body weight of the mice was measured once per week after the first treatment with T cells at day 9. Error bars indicate SEM.

The tumors grew readily in the mice that were treated with CD19 CAR T cells or NT T cells, whereas the mice treated with STEAP1 CAR T cells displayed a substantial inhibition of tumor growth (Figure 6A). There was a statistically significant difference in tumor load between the mice in the STEAP1 CAR group and each of the control groups, as measured by both bioluminescence imaging (Figure 6B) and caliper measurement (Figure 6C). The bioluminescence signals of individual mice at multiple time points are shown in Figure S7. At day 34, the tumors had disappeared in five out of 12 mice in the STEAP1 CAR-treated group. The tumors reoccurred around day 55 in four out of these five mice. By day 60, all mice treated with NT T cells had to be euthanized due to the large size of the tumors (Figure 6D), and only three CD19 CAR-treated mice were still alive. The mice treated with STEAP1 CAR T cells had a significantly extended survival compared with both control groups, with 10 out of 12 mice still alive 60 days after tumor cell engraftment, and five of these mice still alive at the end of experiment (day 85; Figure 6E). These data establish the therapeutic efficacy of STEAP1 CAR T cells *in vivo*.

To assess potential side effects of the CAR treatment, the mice were monitored for body weight and general well-being and activity. No change in their well-being or behavior was observed. There was a slight drop in body weight 7 days after the first round of treatment (Figure 6E). However, this was observed in all three groups, which indicated that the weight loss was due to the adoptive cell transfer process and/or rhIL2, and not specifically due to the STEAP1 CAR T cells. The weights in all CAR T-treated groups returned to pre-therapy levels after 7 days, and no further weight deviations were observed.

### CAR T cell survival and tumor infiltration *in vivo*

**Figure 7:**
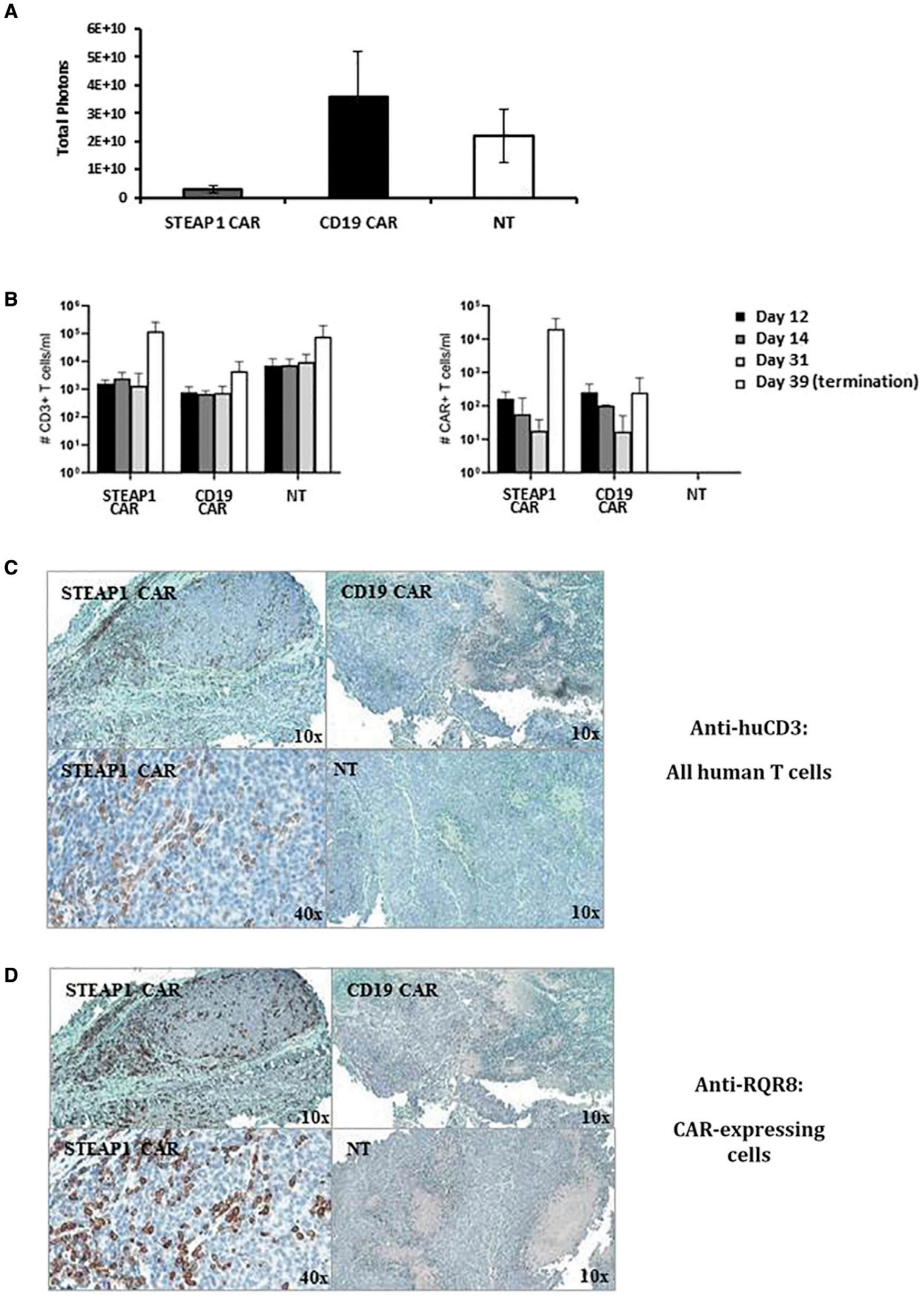
STEAP1 CAR T cells expanded *in vivo* and infiltrated STEAP1+ tumors NSG mice were engrafted subcutaneously with 2 × 10^6^ luciferase-expressing 22Rv1 prostate cancer cells subcutaneously on the hind leg. On day 10 and 17, the mice were treated i.v. with 1 × 10^7^ STEAP1 CAR T cells (N = 3), CD19 CAR T cells (N = 2), or NT T cells (N = 3), and 100 IU/g body weight rhIL-2 was given intraperitoneally twice a week. Mice were sacrificed 39 days after tumor engraftment. (A) Tumor burden assessed by bioluminescence imaging (mean ± SEM) in the eight mice 38 days after tumor engraftment. (B) Peripheral blood cells were measured by flow cytometry at day 12, 14, 31, and 39, and quantified by BD Trucount beads. Left: T cells, identified by anti-human CD3. Right: CAR-expressing T cells, identified by the RQR8-specific mAb QBen10. (C and D) Tumors were fixed, paraffin-embedded, and stained for IHC with anti-huCD3 (T cells) or QBen10 (CAR/RQR8-expressing cells). (C) aCD3 and (D) aRQR8, showing representative staining from each group of mice, with 10× and 40× magnification, as indicated. For the STEAP1 CAR group, tumors from two out of three mice could be stained; the third tumor had completely regressed. T cell infiltration (C) was only observed in the STEAP1 CAR T cell-treated tumors, and corresponded to CAR T cell infiltration (D).

We conducted further experiments to investigate the infiltration of CAR T cells into tumor and normal tissues, and the survival of T cells *in vivo*. The tumor and normal tissues (lung, kidney, liver) were assessed by immunohistochemistry (IHC), while peripheral blood cells were analyzed by flow cytometry. No T cell infiltration into normal tissues was detected (data not shown), whereas overall STEAP1 CAR T infiltration was detected in the tumors of mice treated with the STEAP1 CAR. A representative experiment is shown in Figure 7. Here, eight NSG mice were engrafted s.c. with 22Rv1 prostate cancer cells, and treated with i.v. injection of STEAP1 CAR T cells (N = 3), CD19 CAR T cells (N = 2), or NT T cells (N = 3). The anti-tumor efficacy was assessed by IVIS. As shown in Figure 7A, there was a reduction in tumor burden in the STEAP1 CAR T group, compared with controls. One of the STEAP1 CAR mice had no detectable tumor at study termination. Figure 7B depicts the T cell counts in peripheral blood, quantified with BD Trucount beads at four time points (days 12, 14, 31, and 39). We measured >5-fold higher levels of NT cells at the three first time points, compared with both CAR T groups. At study termination, the T cell counts in the STEAP1 CAR group had increased about 100-fold, and were in line with the NT group. About 20% of the T cells in peripheral blood in the STEAP1 CAR group expressed the CAR construct at this time point, as assessed by the marker RQR8. In the CD19 CAR T group, the percentage of T cells expressing the CAR construct decreased from about 50% at day 12 to 5%–10% at the later time points. The T cell infiltration into tumor was assessed by IHC-staining for human CD3 (all T cells) or the CAR marker protein RQR8. We demonstrated T cell infiltration into the tumor in both STEAP1 CAR-treated mice that had a remaining tumor lesion, but not in any of the five mice that received CD19 CAR or NT T cells (Figure 7C). In contrast to the peripheral blood data, nearly all tumor-infiltrating T cells expressed the CAR construct (Figure 7D).

### STEAP1 CAR T cells effectively target metastatic prostate cancer *in vivo*

In order to investigate the efficacy of STEAP1 CAR T cells targeting metastatic cancer, we established a metastatic prostate cancer mouse model. NSG (J-NXG) mice were injected i.v. with luciferase-tagged 22Rv1 cells, or with STEAP1-knockout 22Rv1 cells, called C11, generated by CRISPR-Cas9-mediated gene editing. Pilot experiments showed that the mice developed large liver metastases, as well as lesions in other organs (data not shown). The C11 cells grew faster *in vivo* than the wild-type 22Rv1 cells when injected i.v. Flow cytometry analyses confirmed that STEAP1 was not expressed in C11 (Figure S8).

**Figure 8:**
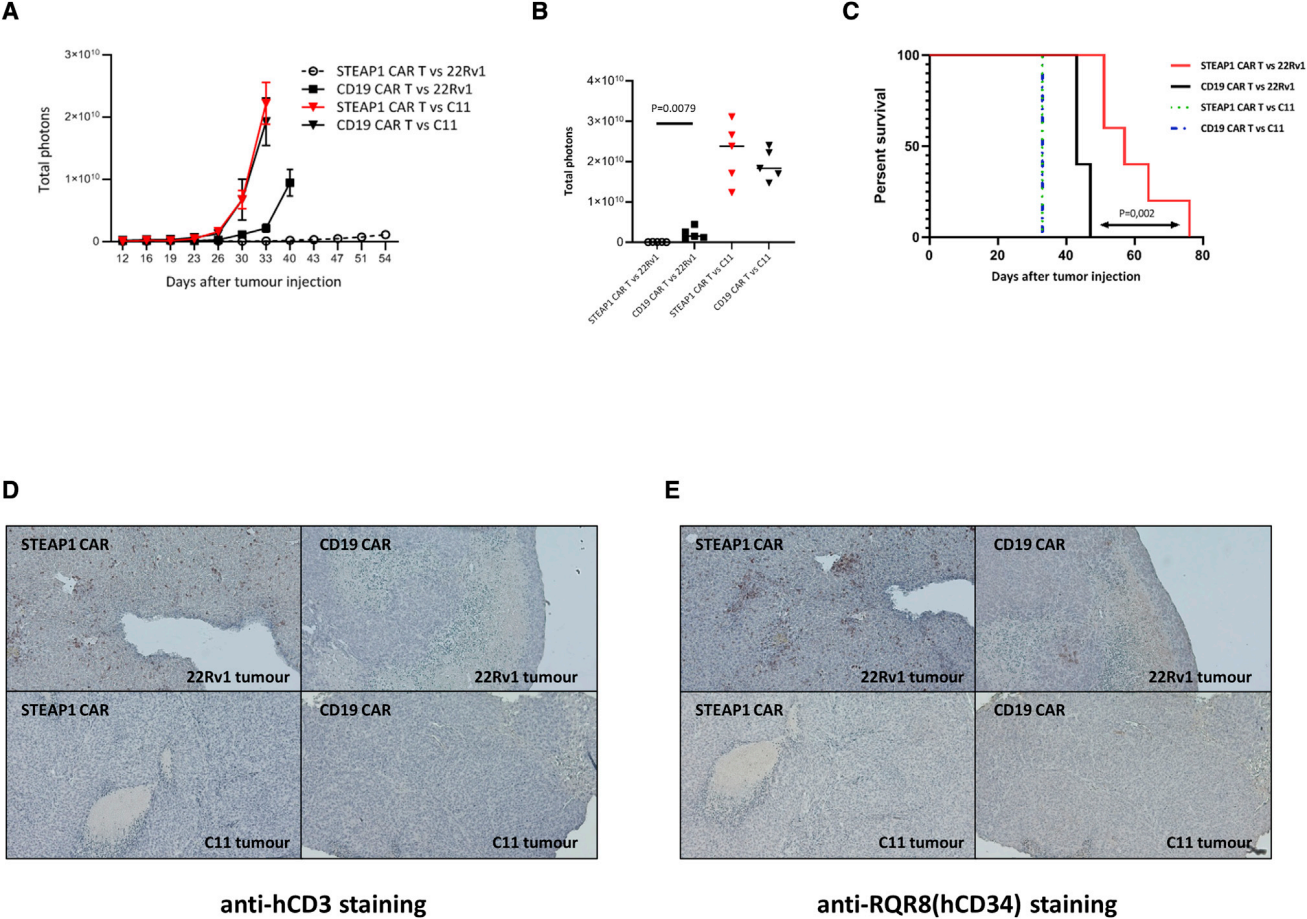
CAR T cells inhibit tumor growth in metastatic *in vivo* model in a STEAP1-dependent manner NSG mice were injected i.v. into the tail vein with 10 × 10^6^ luciferase-expressing 22Rv1 wild-type cells, or with 22Rv1 STEAP1 knockout cells (C11). On day 19 and 26, the mice were treated i.v. with 10 × 10^6^ STEAP1 CAR T cells (N = 5), or CD19 CAR T cells (N = 5), and 100 IU/g body weight rhIL-2 was given intraperitoneally twice a week. (A) Tumor growth was measured once or twice a week by bioluminescence IVIS imaging. The data are presented as mean ± SEM in each group of mice. (B) Bioluminescence signals 33 days after tumor injection. The signal for each mouse is indicated. Statistical analyses were performed using Mann-Whitney U test. (C) Kaplan-Meier plot showing extended survival for mice injected with wild-type 2Rv1 and treated with STEAP1 CAR T cells (red line), compared with CD19 CAR T cells (black line), and to mice injected with 22Rv1 knockout cells (C11; dotted lines). Statistical analysis was performed with log rank test. The mice were euthanized when required by animal welfare guidelines, which generally corresponded to a total photon signal of 1 × 10^10^ (p/s). (D and E) Tumors were fixed, paraffin-embedded and stained for IHC with anti-huCD3 (T cells) or QBen10 (CAR/RQR8-expressing cells). (D) aCD3 and (E) aRQR8, showing a representative staining from each group of mice, with 10× magnification. T cell infiltration (D) was only observed in the STEAP1 CAR T cell-treated tumors and corresponded to CAR T cell infiltration (E).

The 22Rv1/C11 metastatic mouse model was used to further assess *in vivo* CAR T efficacy. NSG mice were injected i.v. at day 1 with 22Rv1 cells or C11 cells. Tumor growth was monitored by bioluminescence imaging. CAR T cells were injected i.v. at days 19 and 26. The STEAP1 CAR T cells showed a strong and statistically significant anti-tumor efficacy, compared with the control CD19 CAR T cells (Figures 8A and 8B). This effect was STEAP1 specific, as it was only observed for the 22Rv1 cells. For mice engrafted with the STEAP1-knockout C11 cells, there was no difference in tumor growth between the STEAP1 CAR T- and CD19 CAR T-treated groups. The STEAP1 CAR T treatment furthermore conferred improved survival, compared with treatment with CD19 CAR T cell controls. The survival effect was dependent on STEAP1 expression in the tumor cells, as it was not observed for C11 mice.

Bioluminescence imaging of individual mice are shown in Figure S9. The 10 mice with C11 tumors all developed large liver metastases and had to be euthanized at day 33. The five 22Rv1 mice treated with CD19 CAR T developed liver metastases by day 41 (Figure S9) and had to be euthanized at day 43–47 (Figure 8C). By contrast, no tumor signal was detected from the liver region in any of the mice in the STEAP1 CAR 22Rv1 group until day 54, and the last mouse in this group survived until day 76 (Figure 8C). Two out of five mice in the STEAP1 CAR 22Rv1 group developed brain metastasis (Figure S9).

To investigate T cell infiltration, the liver tumors were assessed by IHC. The samples were stained for CD3 and the CAR marker RQR8. We observed T cell infiltration into the tumor only in mice injected with wild-type 22Rv1 and treated with STEAP1 CAR T cells, not in the other three mouse groups (Figure 8D). The infiltrating T cells generally stained positive for the CAR marker RQR8 (Figure 8E).

## Discussion

We have developed a CAR against STEAP1. This is a second-generation CAR that includes a 4-1BB co-stimulatory domain. The STEAP1 CAR expression was robust, as the CAR was consistently well expressed across different PBMC donors, and multiple virus productions. In the clinical setting, the logistics require that CAR T cells are cryopreserved until administration to patients. All assays shown here were therefore performed on T cells that were cryopreserved and thawed before functionality testing. Our data show that the STEAP1 CAR conferred transduced T cells with potent functionality, as measured both by the production of multiple cytokines and by the killing of target cells. Repeated exposure to STEAP1+ targets over 3 weeks induced durable CAR T proliferation and a gradual differentiation into EM T cells, with upregulation of checkpoint molecules. Both CD4+ and CD8+ T cells acquired the desired STEAP1-specific activity. Although comparison of CARs against different targets should be interpreted with caution, it is encouraging that the potency of the STEAP1 CAR in *in vitro* assays matched the activity of the CD19 CAR that has an identical design to tisagenlecleucel (Kymriah), which is in clinical use and highly effective in advanced cancer patients.^33^

The results indicate that the STEAP1 CAR has a high specificity. There was strong *in vitro* activity against all STEAP1-positive target cell lines tested, and no detectable reactivity against STEAP1-negative targets. Furthermore, both cytokine secretion and killing were effectively attenuated by shRNA knockdown of STEAP1 in target cells. The Western blot and qPCR data indicated that the relative knockdown efficiency was slightly less in C4-2B than LNCaP cells. Consistent with this, a low/moderate CAR activity was detected in the TNFα and IFNγ assays with the two knockdown C4-2B cell lines. CAR reactivity against low levels of the antigen may be a potential safety risk as STEAP1 CAR T cells could target normal tissues expressing low levels of STEAP1. On the other hand, it may be an advantage in heterogeneous tumors, with zones with differing levels of antigen expression.

The cytokine profile is likely to depend on the T cell expansion protocol, as well as the donor, the CAR, and other factors. In the multiplex assays, we observed a Th1-weighted cytokine profile, which is considered beneficial for anti-tumor responses. There were only minimal levels of the T-regulatory 1-associated cytokine IL-10. Moreover, we detected secretion of multiple chemokines, which may be of importance in transforming the tumor milieu and attracting other immune cells. At the single-cell level, the flow cytometry assays demonstrated simultaneous production of TNFα and IFNγ by individual CAR T cells. In all, these results suggest that the applied T cell expansion and CAR-transduction protocol yielded T cells with a polyfunctional and Th1-weighted cytokine profile for the CAR tested herein. These findings should be confirmed *in vivo*, and with cells produced from patients and with a clinical grade protocol.

The animal data indicated that STEAP1 CAR T cells retained their anti-tumor activity *in vivo*, and exerted tumor control. This applied to both a subcutaneous and a metastatic prostate cancer model, and indicates that the i.v.-injected CAR T cells are capable of migrating to tumors in different locations. The data from the metastatic model are of particular relevance, as a clinical application of STEAP1 CAR T cells would be in metastatic patients. The results in the subcutaneous model were consistent between bioluminescence imaging and caliper measurements, which were applied in parallel for robust efficacy assessment. The antigen-specific effect of the STEAP1 CAR was demonstrated, as tumor control was not detected with a CD19 CAR T control that has an identical co-stimulatory domain and backbone. Furthermore, the experiments with the STEAP1 knockout 22RV1 line confirmed that the *in vivo* efficacy was dependent on STEAP1 tumor expression. The studies were conducted with 22Rv1 prostate cancer cells that have variable levels of expression of STEAP1. These data are encouraging regarding the potential clinical use in patients, where it must be assumed that not all cancer cells express homogeneous and high levels of STEAP1.

Interestingly, we found that the STEAP1 CAR T cells infiltrated the liver metastases, while the CD19 CAR T cells and NT T cells did not. The same pattern was observed in the s.c. tumor model. Furthermore, the T cells expressing the STEAP1 CAR appeared to preferentially home to or expand in the tumor, as nearly all T cells in the tumor expressed RQR8, while the majority in the peripheral blood did not. The number of STEAP1 CAR T cells in peripheral blood increased >100-fold at the latest monitored time point, 4 weeks after first T cell injection. This observation, along with the tumor infiltration data, indicates an ability of these CAR T cells to survive, expand, and infiltrate tumor *in vivo*. The mice receiving NT cells recorded the highest numbers of T cells in the peripheral blood. This may reflect that the STEAP1 CAR T cells migrated to tumor, and that NT cells have a growth advantage, as often observed with cells not subjected to transduction and ectopic gene expression. In spite of the high NT cell numbers in blood, there was no tumor homing of these cells.

To our knowledge, this is the first report on the development of a STEAP1 CAR. There have been various attempts to develop other STEAP1-targeting drugs.^20^^,^^35^, ^36^, ^37^, ^38^ Favorable safety data from a clinical trial with an antibody-drug-conjugate suggests that it is feasible to take a STEAP1-targeting CAR into phase-1 testing.^20^ However, CAR T cell therapy generally carries a higher risk of adverse side effects, compared with antibody-based approaches.^39^ A clinical trial with a bispecific T cell engager (BiTE) targeting STEAP1 is ongoing (NCT04221542). The BiTE approach represents off-the-shelf therapy and has a safety advantage compared with CAR T cells, as the BiTEs are removed from circulation after days or weeks.^40^ On the other hand, CAR T cells are less reliant on the inherent functionality of host T cells, and the clinical experience from CAR T cells and BiTEs against leukemia suggests a higher potential for permanent cure from CAR T cell therapy,^41^ even though BiTE therapy may also induce durable remissions.^42^ A number of interesting approaches are being explored that may be combined with a STEAP1 CAR to overcome resistance in solid cancers.^13^^,^^43^

Our animal data should not be assumed to inform for on-target normal tissue toxicity, as the Oslo-1 mAb was derived from a mouse hybridoma and does not recognize the TRAMP-C2 cell line, which reportedly express murine STEAP1.^34^ Regarding off-target toxicity, it is encouraging that we did not observe CAR T cell infiltration into normal tissues, despite the rich infiltration into the tumor, and high T cell numbers in peripheral blood. It is unclear to what extent cytokines from human T cells induce toxicity in NSG mice. However, we have in a previous evaluation of CD19 CAR variants in a xenograft model, in the same NSG mouse strain, observed that the mice rapidly lost weight and developed severe signs of distress, due to CAR T-related off-target toxicity, and associated with a cytokine storm.^44^ In the present study, no such CAR-related weight loss or signs of distress were detected. Adoptive cell therapy may, however, produce severe side effects that are not detectable in mouse models.^45^, ^46^, ^47^, ^48^ In the present study, we have included the RQR8 protein, which allows for the depletion of CAR T cells with the mAb rituximab.^31^ Such depletion may, however, not be complete. Transient CAR expression based on mRNA-transfection may offer a safer route to clinical testing, but this is less well established.^44^^,^^49^

We conclude that the STEAP1 CAR reported here has antigen-specific and potent *in vitro* and *in vivo* functionality. This includes STEAP1-specific target killing and production of multiple cytokines *in vitro*, and potent anti-tumor activity *in vivo*, accompanied by CAR T cell expansion and infiltration into STEAP1^+^ tumors. We consider that the results warrant further development of the STEAP1 CAR for potential clinical use.

## Materials and methods

### Ethical approval

The study was approved by the Norwegian Food Safety Authority and conducted in accordance with institutional guidelines.

### Sequence identification from hybridoma

The hybridoma ATCC PTA-5803 (X120.545.1.1)^29^ was purchased from ATCC. The hybridoma was cultured for production of purified mAb, which was used for staining cell lines for flow cytometry. The mRNA from cell pellets was extracted and reverse transcription was performed to obtain cDNA for the antibody heavy and light chains. The sequence identification was performed in association with Absolute Antibody (Cleveland, UK). The VH and VL sequences were determined by 5′ RACE, by signal peptide and variable domain sequences were identified by comparison with known sequences in the IMGT database. The identified VH sequence was consistent with that expected for a functional VH domain. The subtype and species of the heavy chain was confirmed as mouse IgG2a. The identified VL sequence was consistent with that expected for a functional VL domain. The subtype and species of the light chain was confirmed as mouse kappa.

### Vector construction

A scFv, termed Oslo1, was designed based on the VH and VL sequences identified from hybridoma ATCC PTA-5803. In brief, VH and VL were generated by using PCR separately with a linker in between and cloned into the SFG retroviral vector. Oligonucleotides were purchased from Eurofins Genomics (Ebersberg, Germany). Plasmid MP 14156 was kindly provided by Dr. Martin Pule, University College London, and used as SFG backbone. The anti-human STEAP1-specific scFv was cloned into retroviral SFG vectors between the NcoI and BamHI restriction sites. A plasmid containing the suicide/sorter/marker gene RQR8 was also provided by Dr. Pule. RQR8 was cloned upstream of the CAR with a 2A self-cleaving peptide.

### Cell lines, primary T cell cultures

LNCaP, 22Rv1, C4-2B prostate cancer cell lines, and NALM6 leukemia cell line were cultured in RPMI-1640 (Sigma-Aldrich, Oslo, Norway) with 10% heat-inactivated fetal bovine serum (FBS) (Sigma-Aldrich, Oslo, Norway) and 100 U/mL penicillin/streptomycin (Sigma-Aldrich, Oslo Norway) (complete medium). Phoenix-Ampho HEK cells were grown in DMEM with 10% Hyclone FBS and 100 U/mL penicillin/streptomycin (Sigma-Aldrich, Oslo Norway). PBMCs were isolated from healthy donor buffy coats using Lymphoprep (Axis-Shield, Oslo, Norway) and cultured in complete medium. T cells from PBMCs were activated using plates coated with 1 μg/mL anti-CD3 (clone OKT3, BioLegend, Oslo, Norway) and 1 μg/mL anti-CD28 (clone CD28.6, eBioscience, Oslo, Norway), in addition to 100 IU/mL rhIL-2 (R&D Systems, Abingdon, UK).

### Retroviral and lentiviral transduction

Phoenix-Ampho HEK cells (1.2 × 10^6^ per 6cm dish, 2.8 × 10^6^ per 10-cm dish) were plated overnight before co-transfection of packaging plasmids with the CAR vector using X-tremeGENE 9 reagent (Roche, Oslo, Norway). The supernatant was harvested 2 and 3 days after transfection, frozen, and stored at −80°C. T cells were activated with anti-CD3 plus anti-CD28 antibodies and rhIL-2 (100 IU/mL) for 2 days. The transduction was performed in Retronectin-coated plates (Takara, Gothenburg, Sweden) with 0.3 × 10^6^/mL activated PBMC suspended in 2 mL of complete medium supplemented with rhIL-2 (200 IU/mL) plus 2 mL of retroviral supernatant, centrifuged at 900 × *g* for 60 min at 32°C, and then incubated at 37°C. Two days later, the medium of CAR T cells was changed with fresh complete medium supplemented with rhIL-2 (100 IU/mL) and expanded for three more days. The expanded T cells were frozen and stored in liquid N_2_. Before functional assessment in the *in vitro* or *in vivo* experiments, the T cells were thawed and re-activated for 2 days with anti-CD3 and anti-CD28 antibodies plus rhIL-2 (100 IU/mL). The NT cells were not suspended in a viral supernatant and did not undergo spinoculation. Otherwise, the NT cells were stimulated and expanded under the same conditions as the CAR T cells.

22Rv1 cells were transduced with pLenti CMV V5-LUC Blast (w567-1) (a gift from Eric Campeau; Addgene plasmid # 21474; http://n2t.net/addgene:21474; RRID:Addgene_21474) as described.^50^ 22Rv1 cells expressing nuclear GFP were further transduced with pFU-H2B-GFP-IRES-Puro (a gift from Charles Gersbach; Addgene plasmid # 69550; http://n2t.net/addgene:69550; RRID:Addgene_69550). Lentiviral pLKO1 shRNA vectors targeting human STEAP1 and non-silencing pLKO1 control vector were purchased from Thermo Fisher Scientific, Oslo, Norway. Lentivirus particles were produced in 293T cells according to the developer’s instructions.^51^ LNCaP or C4-2B cells were transduced by the lentiviral particles followed by puromycin selection (1 μg/mL) for 10 days. The cells stably expressing shRNA were pooled and maintained in complete medium with 0.2 μg/mL puromycin.

### Generation of 22Rv1-KO cell line

STEAP1 gene was inactivated in 22Rv1 cell line using CRISPR-Cas9-mediated gene editing. Specifics oligonucleotides to single guide RNAs (sgRNAs) targeting exons 3 or 5 of STEAP1 gene were cloned into the plasmid vector lentiCRISPR v2 (a gift from Feng Zhang, Addgene plasmid # 52961; http://n2t.net/addgene:52961; RRID:Addgene_52961)^52^ using the protocol described by Ran et al.^53^ The sgRNA sequences are as follows: sgRNA 1-Exon 5, 5′-TGTATTGTGCCCAGTAGAA-3′; sgRNA 2-Exon 5, 5′-CGTGTATTGTGCCCAGTAGA-3′; sgRNA 3-Exon 3, 5′-CAATTGTCCAACTTCATAA-3′. 22Rv1 cells were transfected by STEAP1-sgRNAs LentiCRISPR v2 virus in presence of 4 μg/mL of polybrene (Sigma-Aldrich, Oslo Norway). Forty-eight hours after transduction, cells were selected for 5 days with 1 μg/mL puromycin and subcloned by limited dilutions. Up to 30 clones from each sgRNA were screened by flow cytometry and cells lacking STEAP1 expression were analyzed for indel mutation by next-generation sequencing (NGS) (Eurofins Genomics). One knock out 22Rv1 clone (clone C11, sgRNA 2-Exon 5) was used for experiments.

### qPCR

RNA was extracted using TRI Reagent Solution. The cDNA synthesis was performed with SuperScript IV Reverse Transcriptase. The qPCR was run on the StepOnePlus Real-Time PCR System using Fast SYBR Green Master Mix and the following primers: STEAP1 forward, GGCGATCCTACAGATACAAGTTG; and STEAP1 reverse, CAGCCAACAGAGCCAGTATT. TATA-Box-binding protein (TBP) was used as internal reference control (forward, GAGAGTTCTGGGATTGTACCG; reverse, ATCCTCATGATTACCGCAGC). All reagents and equipment were purchased from Thermo Fisher Scientific, Oslo Norway. The experiments were performed in triplicate with consistent results.

### Western blot analysis

Cells were washed with ice-cold PBS and lysed in sample buffer (125 mM Tris-HCl, pH 6.8, 4% SDS, 20% glycerol, 200 mM DTT, and 0.004% bromophenol blue). Cell lysates were then subjected to SDS-PAGE on 4%–20% (Bio-Rad Norway AS. Oslo, Norway) gradient gels and blotted onto Immobilon-P membranes (Sigma-Aldrich, Oslo Norway). Membranes were incubated with goat anti-human STEAP1 antibody (sc-10262; Santa Cruz Biotechnology, Heidelberg, Germany) or mouse anti-human vinculin monoclonal antibody (Sigma-Aldrich, Oslo, Norway) at 4°C overnight. Next, the membranes were washed and incubated with the fluorescently labeled secondary antibodies IRDye 680RD donkey anti-goat immunoglobulin G (IgG) and IRDye 680RD donkey anti-mouse IgG (LI-COR, Homburg, Germany) and analyzed by Odyssey infrared scanner (LI-COR, Bad Homburg, Germany).

### Flow cytometry T cell assays

The CAR and NT T cells were then frozen after expansion and thawed before use in functional T cell assays. For flow cytometry cytokine assays, the T cells were co-cultured with various target cells at an E:T ratio of 1:3 for 16 h in the presence of BD GolgiStop and BD GolgiPlug (BD Biosciences, Franklin Lakes, NJ, USA). IFNγ and TNFα production in the total CD3+ T cell- or RQR8-positive population was determined by flow cytometry.

CAR T cell cytotoxicity was assessed by co-culturing the T cells with target cells at the specified E:T ratios for 16 h and assessing target cell caspase activation and cell death by flow cytometry. Caspase-3 activation in the target cells was measured by the addition of fluorescein isothiocyanate (FITC)-DEVD-FMK (Thermo Fisher Scientific, Oslo, Norway) from the start of the co-culture. Cell death was detected by the eBioscience Fixable Viability Dye eFluor 780 (Thermo Fisher Scientific, Oslo, Norway).

### Flow cytometry instruments and reagents

Flow cytometry analysis was performed on LSR II, Fortessa or BD Symphony flow cytometers (BD Biosciences, Franklin Lakes, NJ, USA) and data analyzed with FlowJo software (Tree Star, Ashland, OR, USA). The following antibodies and reagents were used: CD3-Pacific Blue (clone OCT3), CD3-PerCP-Cy5.5 (clone OCT3) CD8-BV711, CD8-BUV563 (clone RPA-T8), CD4-BV510 (clone SK3), CD25-BV605 (clone BC96), TIM3-BV711 (clone F38-2E2) CCR7-AF594 (clone G043H7), TIGIT-BV421 (clone A15153G), and LAG3-PE.Cy7 (clone 11C3C65) (BioLegend, Oslo, Norway); CD45RA-BUV496 (clone 5H9), PD-1-BV786 (clone EH12.1), and TNF-PE (clone Mab11) (BD Biosciences Nordic, Oslo, Norway); CD19-PE (clone 4G7-3E3; R&D Systems, Abingdon, UK); IFNγ-PE-Cy7 (clone 4S.B3), donkey anti-mouse IgG AF568 (catalog no. A10037) and Fixable Viability Dye efluor780 (Thermo Fisher Scientific, Oslo, Norway); goat anti-mouse IgG AF488 (catalog no. 115-545-071) and goat anti-rabbit IgG AF488 (catalog no. 11-546-046; Jackson Immuno Research, PA, USA). Expression of the STEAP1 CAR or the CD19 CAR was determined using the RQR8-binding mAbs QBend10-PE or QBend10-APC (R&D Systems). Target cell apoptosis was determined by CaspGLOW Fluorescein Active Caspase-3 Staining Kit (Thermo Fisher Scientific, Oslo, Norway). Intracellular staining was performed using the BD Cytofix/Cytoperm reagent, according to the manufacturer’s protocol (BD Biosciences Nordic, Oslo, Norway).

### Longitudinal analysis of CAR T cell proliferation and phenotype upon repeated target exposure

The target 22Rv1 cells were transduced with a lentivirus expressing nucleus-located GFP. After cell sorting, more than 98% of 22Rv1 cells expressed GFP. The GFP-22Rv1 target cells were seeded at 1 × 10^6^/well in 24-well plates, cultured for 24 h, and irradiated at 20 Gy. After 1 day, cryopreserved CAR T cells were thawed and added at 1 × 10^6^/well. Half of the suspended cells were transferred to a new 24-well plate seeded with irradiated target cells twice per week, plus the same amount of fresh medium containing 10% FBS. The cells were counted and phenotyped by flow cytometry once per week, using 123count eBeads Counting Beads (Thermo Fisher Scientific) and mAbs for phenotypic markers as indicated. The samples were analyzed on a BD Symphony flow cytometer (BD Biosciences, Franklin Lakes, NJ, USA).

### Multiplex cytokine assay

Cryopreserved PBMCs that had been previously transduced with either the STEAP1 CAR, CD19 CAR, or left NT were thawed and co-cultured with 22Rv1 target cells for 48 h at a 1:1 E:T ratio. The supernatant was collected, and the secreted cytokines and chemokines quantified using the 20-plex Bio-Plex Pro Human Immunotherapy panel (Bio-Rad Norway AS, Oslo, Norway) to the manufacturers protocol. The data were analyzed using the Luminex 100 System (Bio-Rad, Norway AS, Oslo, Norway) and cytokine/chemokine concentrations calculated using Bio-Plex Manager 6.1. Supernatants were analyzed in duplicate, each parallel kept separate through T cell stimulation and Bio-Plex assays.

### Real-time killing assay

The real-time killing assay was performed with IncuCyte S3 live cell image system (Sartorius AG, Goettingen, Germany). The target cells 22Rv1 were transduced by lentivirus to express nucleus-located GFP. The target cells were seeded at 1 × 10^5^/well in 96-well plates and irradiated at 20 Gy. The next day, cryopreserved T cells were thawed and added to the culture at E:T ratio of 5:1 and 2.5:1. The plate was real-time monitored and imaged by IncuCyte S3 every 3 h for 7 days.

### Subcutaneous *in vivo* prostate cancer xenograft studies

NSG (NOD.Cg-Prkdc^scid^ Il2rg^tm1Wjl^/SzJ) (JAX) immunodeficient mice were bred in house, and 6- to 8-week-old mice were subcutaneously injected with 2 × 10^6^ 22Rv1 cells (firefly luciferase transduced) + 20% Matrigel (Corning Life Sciences, Wiesbaden, Germany) on the left flank. When the tumors were palpable (10–12 days post implantation), 1 × 10^7^ T cells per mouse were injected into the tail vein. A second T cell i.v. injection (1 × 10^7^ cells per mouse) was performed at the same time point for all mice in each experiment (5–7 days after the first injection, as indicated), and 100 IU of rhIL-2 per gram body weight were injected intraperitoneally (i.p.) twice per week. Tumor growth was monitored by bioluminescence imaging once per week (Xenogen Spectrum system; Grantham, UK). Briefly, anesthetized mice were injected i.p. with 150 μg/g body weight of D-luciferin (PerkinElmer Norway, Oslo, Norway) and imaged 18 min after luciferin injection. In parallel, the tumors were measured by caliper twice per week.

For assessment and quantification of T cells in peripheral blood, the mice were bled at various time points after therapy administration, via tail vein excision, and the blood collected in Microvette 500 K3 EDTA tubes (Sarstedt AS, Oslo, Norway). Fifty microliters of whole blood was then reverse pipetted into BD Trucount tubes (BD Bioscience, Oslo, Norway) and incubated with CD3-Pacific Blue (BioLegend, Oslo, Norway) and the anti-CD34 antibody QBen10-APC (R&D Systems, Abingdon, UK). The blood was lysed using BD FACS Lysing solution (BD Bioscience, Oslo, Norway) and the samples acquired immediately on a Fortessa flow cytometer and analyzed using FlowJo software (Tree Star, Ashland, OR, USA). The absolute cell counts were calculated by dividing the number of acquired events in the CD3 or CD34 gates by the number of acquired events in the bead gate and multiplying by the number of beads listed on the BD Trucount pouch used.

### Metastatic *in vivo* prostate cancer xenograft studies

NOD.Cg-Prkdc^scid^ Il2rg^tm1Wjl^/SzJ (NXG) (Janvier Labs, Le Genest-Saint-Isle, France) immunodeficient mice were bred in house, and 6- to 8-week-old mice were injected i.v. with 10 × 10^6^ 22Rv1 cells (firefly luciferase transduced) or STEAP1-knockout 22Rv1 (clone C11) into the tail veins. When the bioluminescence signal was detectable (about 20 days post tumor injection), 1 × 10^7^ T cells per mouse were injected into the tail vein. A second T cell i.v. injection (1 × 10^7^ cells per mouse) was performed at the same time point for all mice in each experiment (7 days after the first injection, as indicated), and 100 IU of rhIL-2 per gram body weight were injected i.p. twice per week. Tumor growth was monitored by bioluminescence imaging twice or once per week (Xenogen Spectrum system, Grantham, UK). Briefly, anesthetized mice were injected i.p. with 150 μg/g body weight of D-luciferin (PerkinElmer Norway, Oslo, Norway) and imaged 12 min after luciferin injection.

### IHC

IHC was conducted to assess T cell infiltration in xenograft tumors and normal tissues. In brief, the tissues were fixed with 10% buffered formalin and embedded with paraffin. The sections were deparaffinized before being transferred for epitope retrieval, induced by heat for 20 min. Prior to the antibody incubations, endogenous peroxidase was blocked with BLOXALL Endogenous Blocking Solution (SP-6000-100, BioNordika) for 15 min at room temperature (RT). The sections were then washed and incubated for 2 h with blocking buffer consisting of 10% NGS and 1% BSA in PBS. Subsequently, the tissue sections were incubated at 4°C overnight with primary antibodies against huCD3 (ab17143, Abcam, Cambridge, UK; 1:200), or against the huCD34-epitope in marker gene RQR8 (ab8536, Abcam, Cambridge, UK; 1:500). The slides were washed and incubated in goat anti-mouse peroxidase-conjugated secondary antibody (1:200) at RT for 1 h. Signal was visualized with 3,3′-diaminobenzidine (DAB) chromogen and sections were counterstained with hematoxylin and imaged by Zeiss Axiolab 5 microscope (Zeiss Norway, Oslo, Norway).

### Statistical analysis

Statistical analyses were performed using GraphPad Prism 8. In the flow cytometry T cell assays, the percentage of positive cells recorded for the STEAP1 CAR versus the CD19 CAR was compared by multiple t tests, and the statistical significance determined using the Holm-Sidak method for adjusting for multiple comparisons. For the *in vivo* experiments, Mann-Whitney U test was used for statistical comparisons of tumor load between two groups, and survival was calculated using the Kaplan-Meier method. Statistical comparisons of survival was performed by log rank test. All p values given are two-tailed values. Associations with a p value below 0.05 were considered statistically significant.

## Data Availability

Experimental protocols, data, and material are available upon request.

## Supplemental information

Document S1. Figures S1–S9

Document S2. Article plus supplemental information
