# Supplementary material for: Development of STEAP1 targeting chimeric antigen receptor for adoptive cell therapy against cancer
Source: Mol Ther Oncolytics. 2022 Jun 22;26:189–206. doi: 10.1016/j.omto.2022.06.007 (PMC9278049; doi:10.1016/j.omto.2022.06.007)
Supplement: Document S1. Figures S1–S9 [file mmc1.pdf]

OMTO, Volume *26*

Supplemental information

Development of STEAP1 targeting chimeric antigen receptor for adoptive cell therapy against cancer

Yixin Jin, Kristina Berg Lorvik, Yang Jin, Carole Beck, Adam Sike, Irene Persiconi, Emilie Kvaløy, Fahri Saatcioglu, Claire Dunn, and Jon Amund Kyte

SUPPLEMENTARY FIGURES

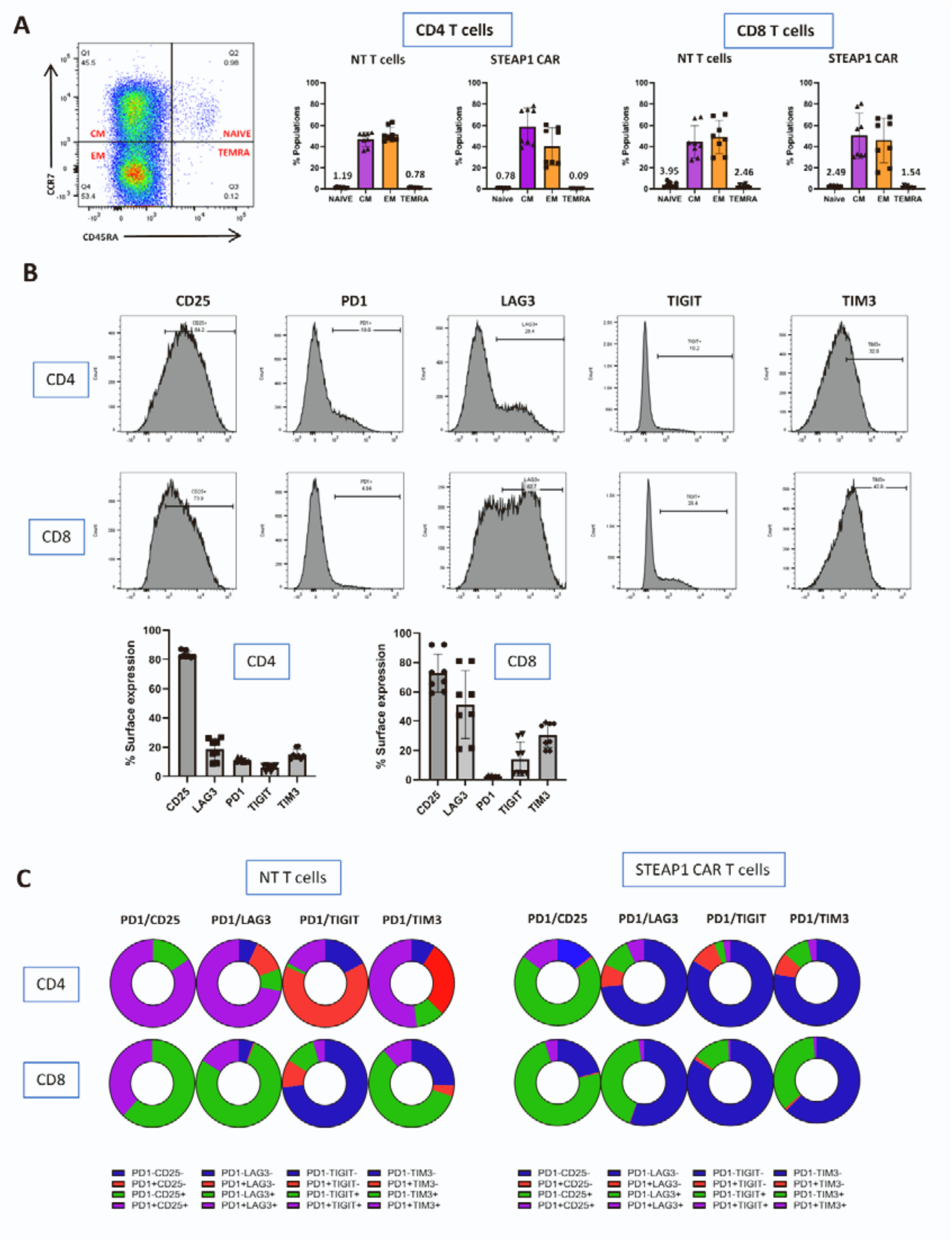

Supplementary Figure S1: STEAP1 CAR T cell phenotyping

PBMC isolated from four healthy donors were stimulated for 2 days with CD3/CD28 antibodies and rhIL-2 before retroviral transduction with the STEAP1-CAR. The cells were cultured for an additional 4 days before phenotyping by flow cytometry (duplicate samples from each donor). (A) Representative dot plot (left) showing CCR7 and CD45RA expression on CAR+ T cells. Bar graphs (right) show the proportion of naive, effector memory (EM), central memory (CM), and T effector memory re-expressing CD45RA (TEMRA) T cell subsets for CAR+ and non-transduced (NT) T cells (duplicates from four donors). (B) $CD4^+$ and $CD8^+$ STEAP1 CAR T cells were assessed for surface expression of the activation marker CD25 and the checkpoint receptors PD1, LAG3, TIGIT & TIM3. Representative histograms indicate the gating and expression levels. Background staining was identified using Fluorescence Minus One controls. Bar charts show a summary of marker expression on $CD4^+$ and $CD8^+$ T cells (duplicates from four donors). (C) 'Parts of Whole' graphs showing the co-expression of PD1 with CD25, LAG3, TIGIT, and TIM3 on CD4+ and CD8+ NT T cells and STEAP1 CAR T cells. Average expression values from four healthy donors were used.

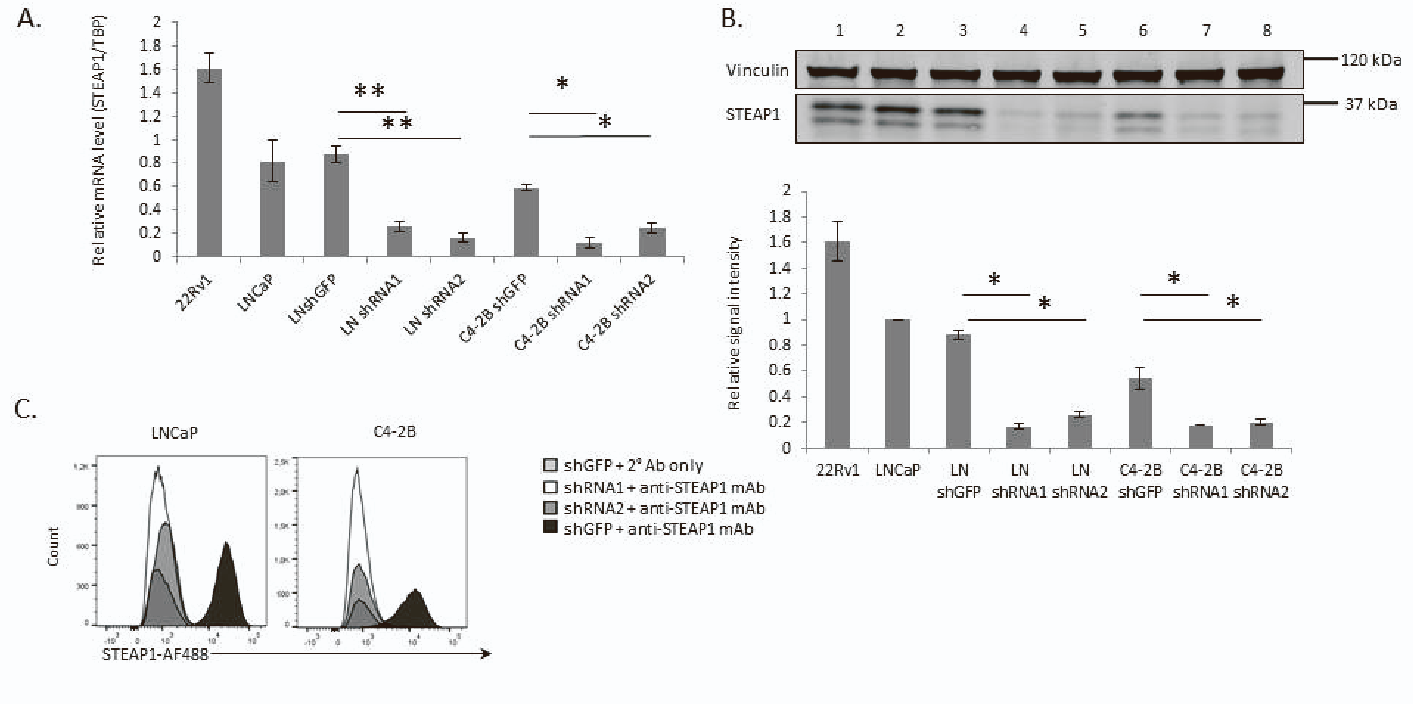

Supplementary Figure S2: STEAP1 expression in prostate cancer cell lines

The expression of STEAP1 was analysed in prostate cancer cell lines 22Rv1 and LNCaP, and in LNCaP and C4-2B cells transduced to express shRNA knocking down STEAP1 (shRNA1, shRNA2) or control shRNA (shGFP). A) Real-time PCR analysis of STEAP1 mRNA expression. TBP was used as reference gene. B) Western blot analysis of STEAP1 expression. Bar chart shows quantification of STEAP1 signal intensity levels compared and normalized to Vinculin in each cell line (n=2). C) Flow cytometry analysis of STEAP1 expression on LNCaP and C4-2B cells treated with STEAP1-targeting shRNA1 (black open) or shRNA2 (grey), or shGFP (black). Cells treated with shGFP stained with the secondary (2nd) Ab only (light grey) served as a staining control. (* P<0.05, ** P<0.01)

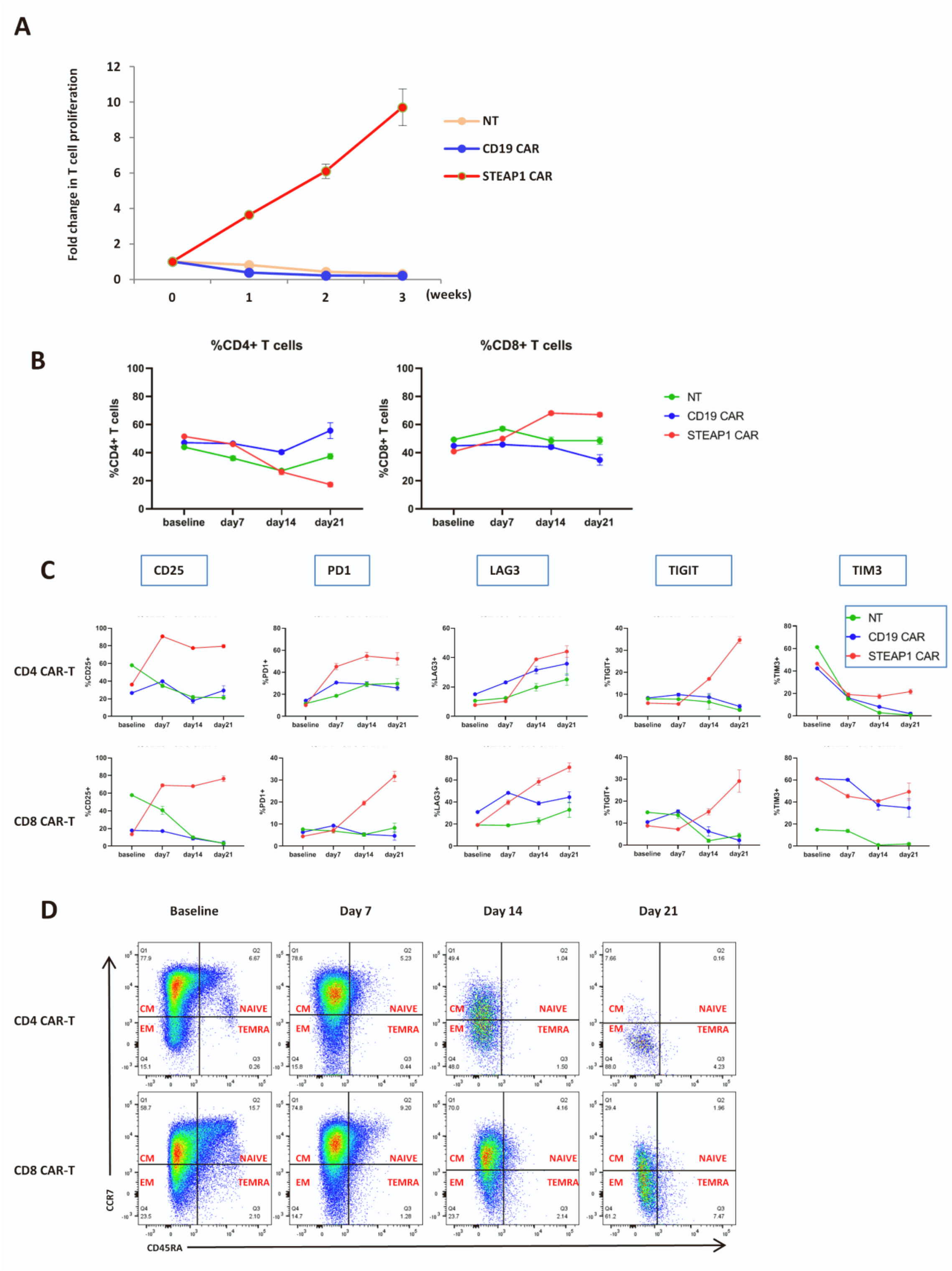

Supplementary Figure S3: Longitudinal analysis of CAR T cell proliferation and phenotype upon long term co-culture with target cells

Irradiated 22RV1 target cells were seeded at $1x10^6$/well in 24-well plates. After 1 day, CAR T cells were thawed and added at $1x10^6$/well. Twice per week, half of the suspended cells were transferred to a new plate seeded with irradiated target cells. The cells were counted and phenotyped by flow cytometry once per week. To separate the cell populations, the 22Rv1 cells had been transduced with GFP and sorted, giving >98% GFP+ 22Rv1 cells. (A) T cell expansion measured by 123count eBeads™ Counting Beads. (B) CD4+ and CD8+ T cell fractions. (C) Expression of CD25, PD1, LAG3, TIGIT & TIM3. Background staining was identified using Fluorescence Minus One controls. (D) Maturation subsets of STEAP1 CAR T cells at different time points: Naive, effector memory (EM), central memory (CM), T effector memory re-expressing CD45RA (TEMRA). Error bars represent SEM of quadruplicates.

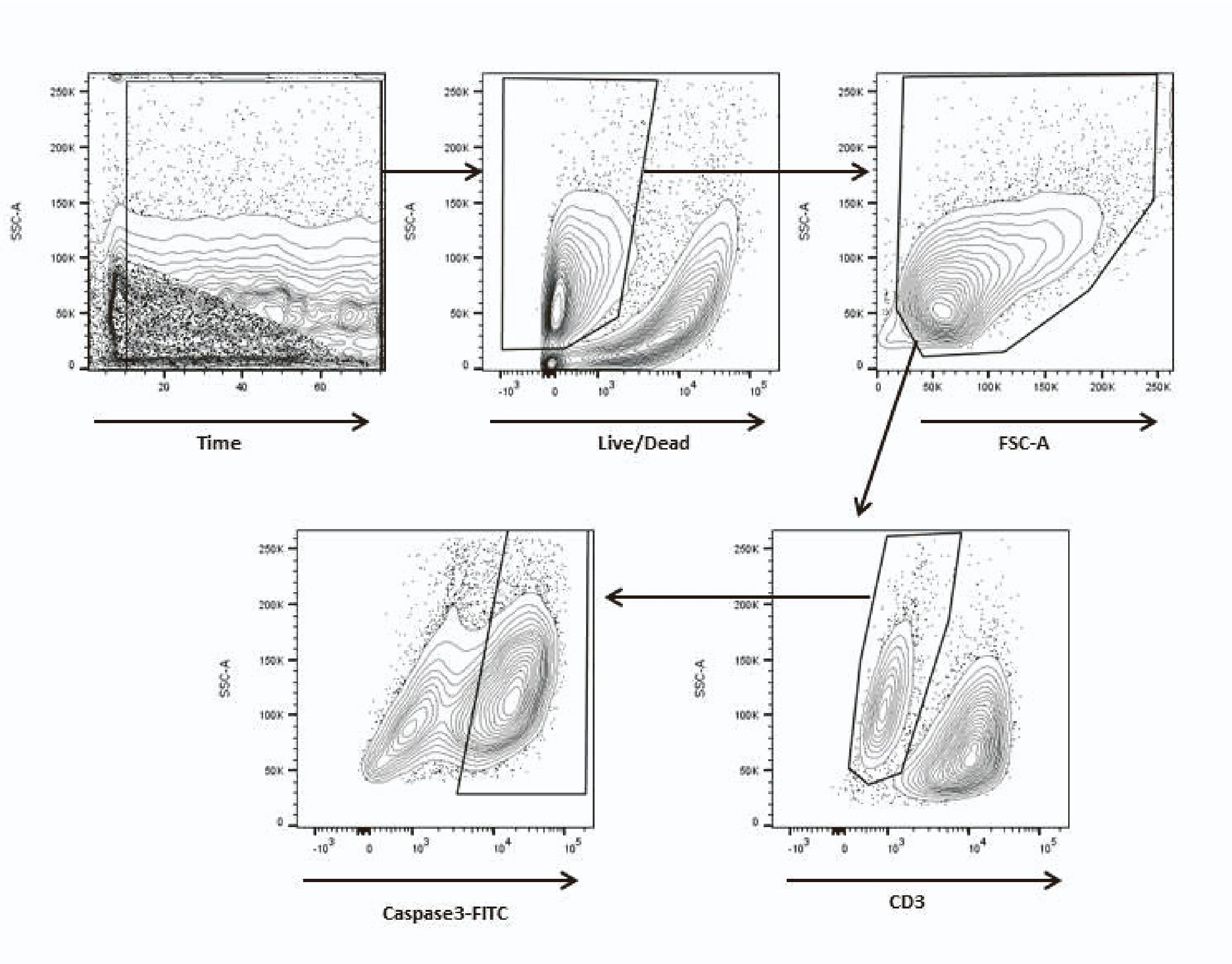

Supplementary Figure S4: **Gating strategy of apoptotic target cells**
Apoptosis of target (tumour) cells was measured by analysing the intensity of FITC-DEVD-FMK bound to active caspase-3 by flow cytometry. The percentage of apoptotic tumour cells was calculated after gating out the dead cells and the effector T cells. Dead cells were excluded with Fixable Viability Dye eFluor™ 780. Effector T cells were excluded by CD3 staining.

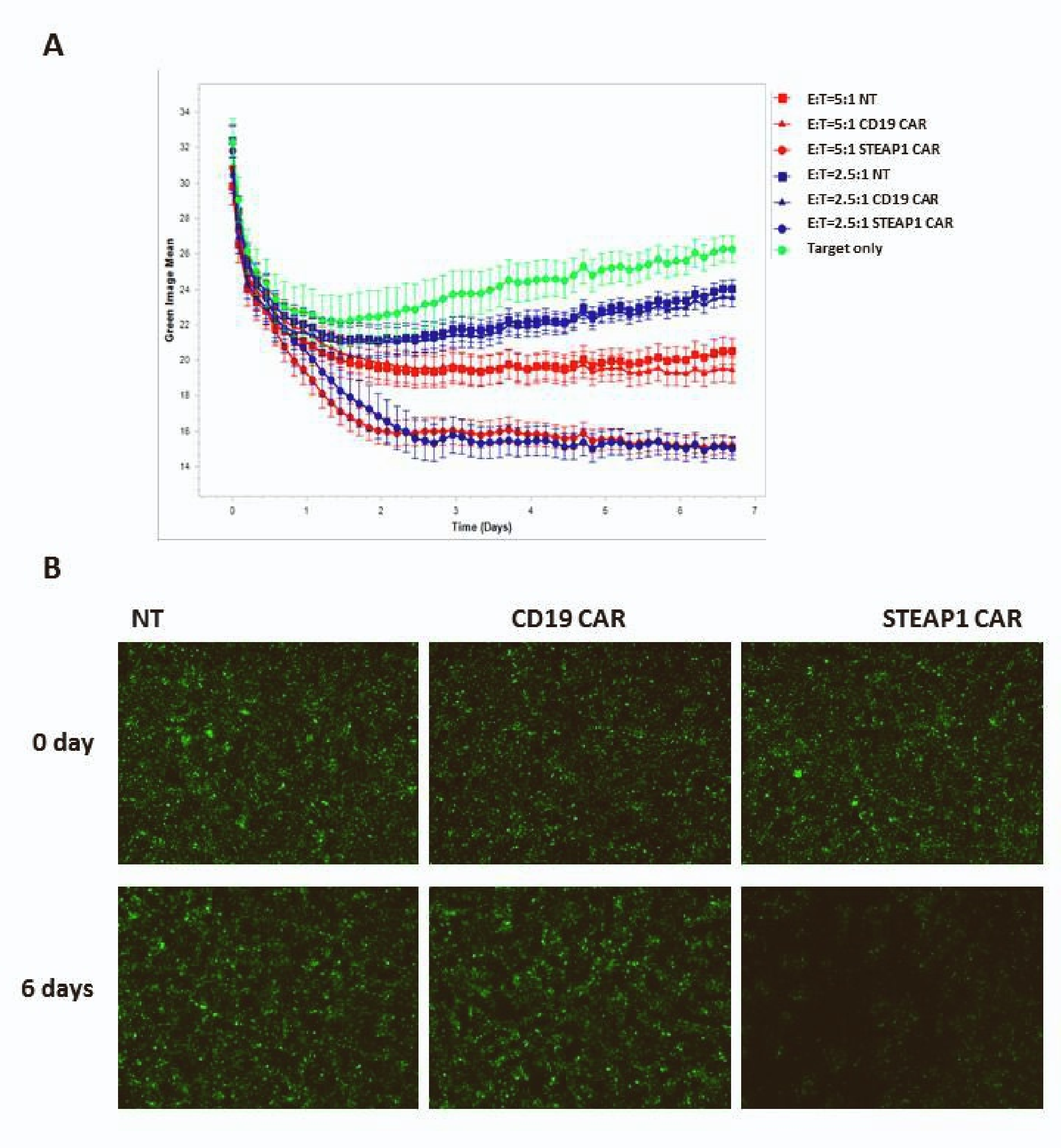

Supplementary Figure S5: **Real time monitoring of target killing by STEAP1 CAR T cells**

The target cells 22Rv1 were transduced by lentivirus to express the nucleus-located protein GFP. One day before coculture with CAR-T cells, 22Rv1 target cells were seeded at $1x10^5$/well in 96-well plates and irradiated at 20Gy. Cryopreserved T cells were thawed and added to the target cells at the indicated E:T ratios. The plate was real-time monitored over 7 days and imaged by IncuCyte S3. (A) Mean GFP expression was determined by the IncuCyte S3 built-in software. (B) Representative images showing GPF+ 22Rv1 target cells at the start of the coculture and on day 6 (E:T=2.5:1).

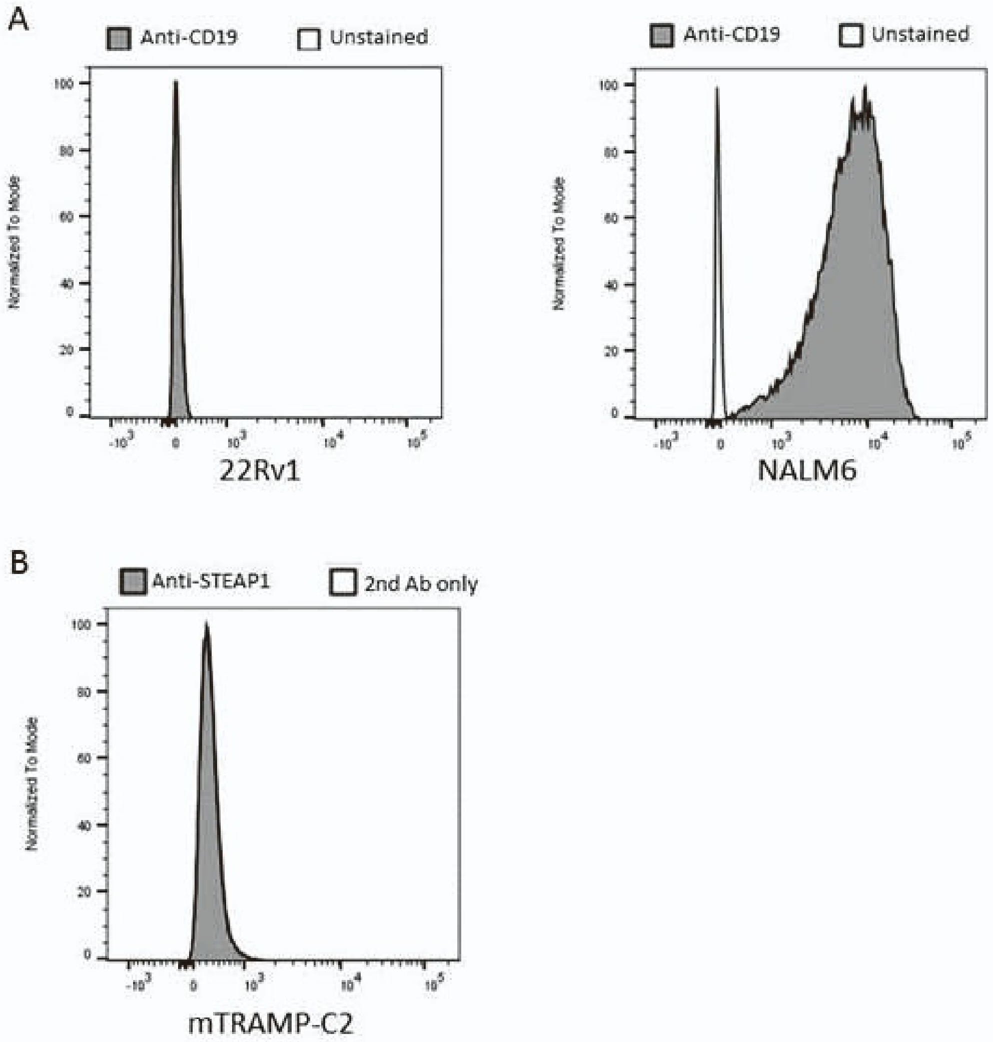

Supplementary Figure S6

A. Prostate cancer cell line 22RV1 did not express CD19. 22Rv1 cells and NALM6 cells (CD19+ leukemia cells) were stained with anti-CD19 PE and analysed by flow cytometry.

B. The anti-STEAP1 mAb used for CAR-development showed no cross-reactivity with murine STEAP1. Murine prostate cancer cell line TRAMP-C2, which expresses murine STEAP1, was stained with the anti-STEAP1 mAb and a secondary Ab, and analysed by flow cytometry.

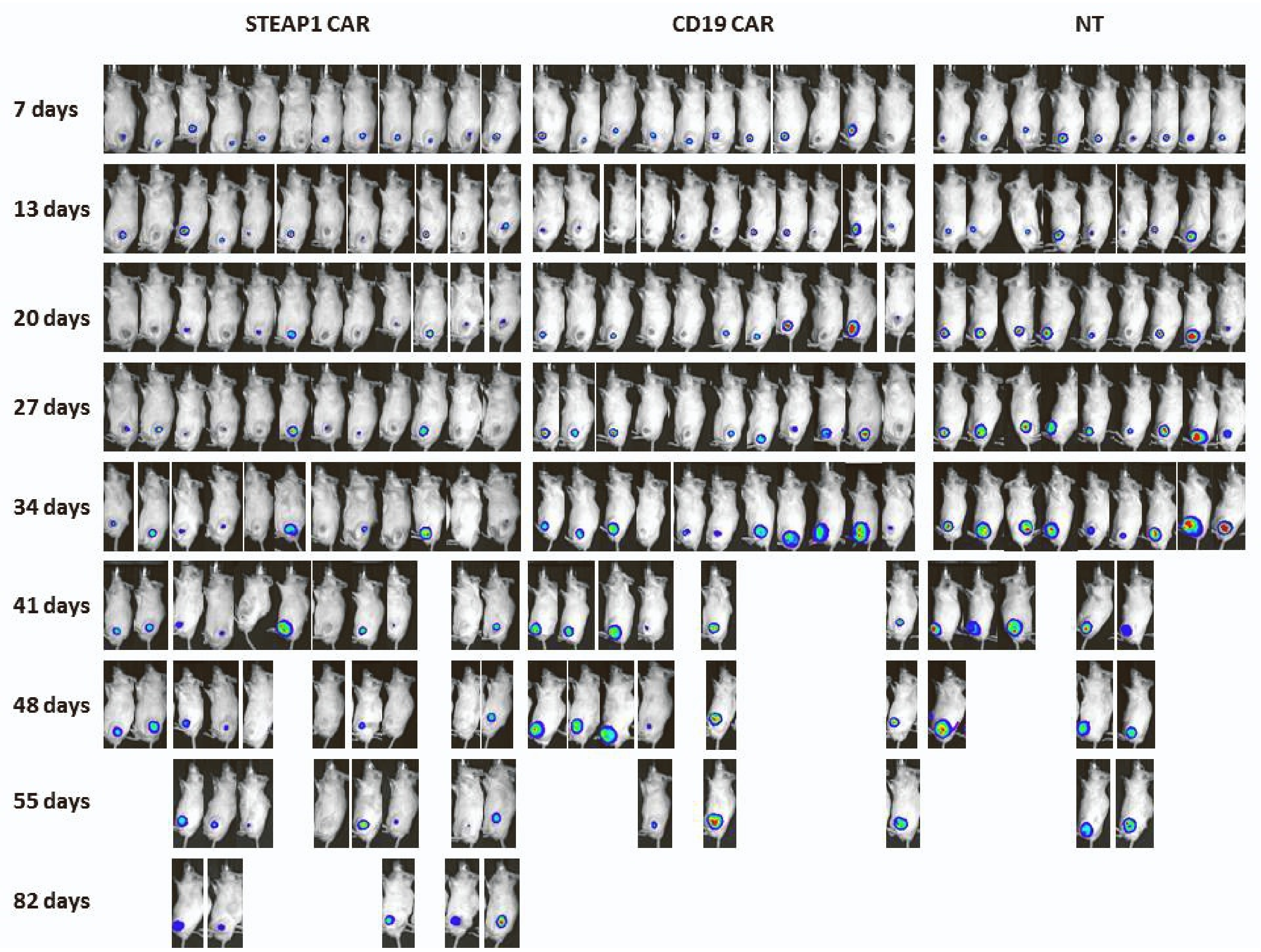

Supplementary Figure S7: Bioluminescence images of individual mice at multiple time points in subcutaneous model experiment

Images from the experiment shown in Figure 6. NSG mice were engrafted subcutaneously on the hind leg with $2x10^6$ luciferase expressing 22Rv1 prostate cancer cells. On day 9 and 14, the mice were treated with $1x10^7$ STEAP1 CAR T cells (N=12), CD19 CAR T cells (N=11) or non-transduced T cells (N=9) by intravenous injection Bioluminescence signals of individual mouse were measured at indicated time points (days after tumour injection).

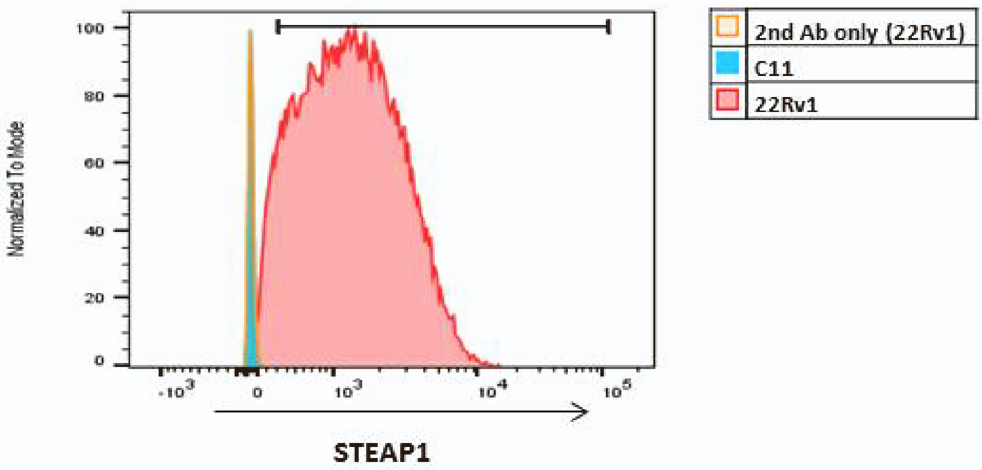

Supplementary Figure S8: STEAP1 expression in 22Rv1 wildtype and STEAP1 knockout cell line
Wildtype 22Rv1 cells and clone C11 22Rv1 STEAP1 knockout cells were stained for STEAP1 expression by use of the Oslo1 anti-STEAP1 mAb and a secondary Ab (donkey anti mouse IgG AF568), and analysed by flow cytometry. Orange/blue filled histograms: anti-STEAP1 mAb + secondary (2nd) Ab; Orange open histogram: Secondary Ab control for 22Rv1 cells.

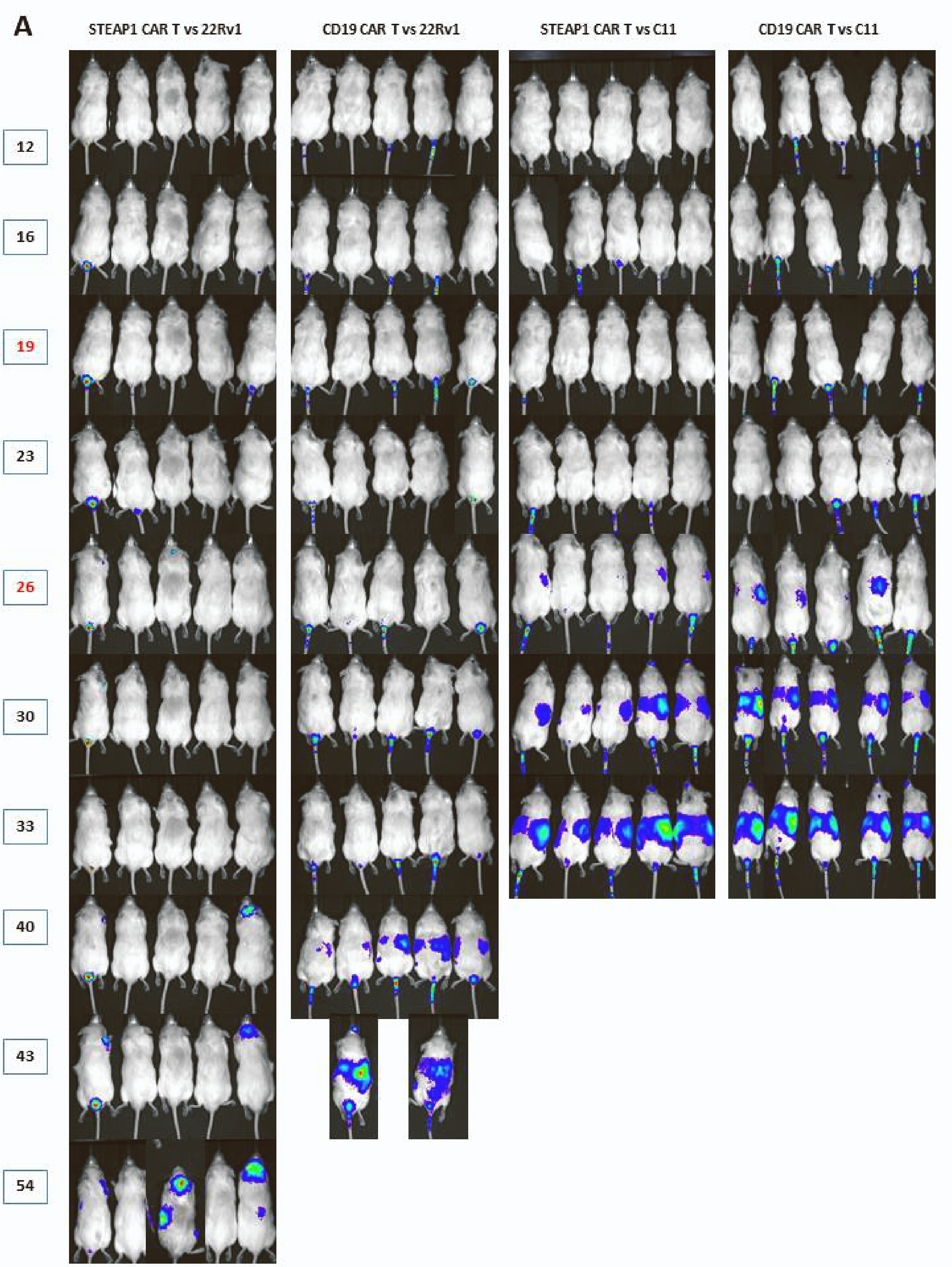

Supplementary Figure S9: Bioluminescence images at of individual mice in metastatic model
Images from the experiment ishown in Figure 8. NSG mice were injected i.v. into the tail vein with $10x10^6$ luciferase expressing 22Rv1 wildtype cells, or with 22Rv1 STEAP1 knock-out cells (C11). On day 19 and 26, the mice were treated i.v. with $10x10^6$ STEAP1 CAR T cells (N=5), or CD19 CAR T cells (N=5). Tumour growth was measured by bioluminescence IVIS imaging at the indicated time points (days post tumour injection). The time points for T cell injections are indicated in red.
